# Lipid profile migration during the tilapia muscle steaming process revealed by a transactional analysis between MS data and lipidomics data

**DOI:** 10.1038/s41538-021-00115-1

**Published:** 2021-11-15

**Authors:** Rui Sun, Tingting Wu, Hao Guo, Jiamin Xu, Jiahui Chen, Ningping Tao, Xichang Wang, Jian Zhong

**Affiliations:** 1 National R & D Branch Center for Freshwater Aquatic Products Processing Technology (Shanghai), Integrated Scientific Research Base on Comprehensive Utilization Technology for By-Products of Aquatic Product Processing, Ministry of Agriculture and Rural Affairs of the People’s Republic of China, Shanghai Engineering Research Center of Aquatic-Product Processing and Preservation, College of Food Science & Technology, Shanghai Ocean University, Shanghai, 201306 China; 2 Chongqing Institute of Forensic Science, Chongqing, 400021 China; 3 Collaborative Innovation Center of Seafood Deep Processing, Dalian Polytechnic University, Dalian, 116034 Liaoning Province China

**Keywords:** Lipidomics, Mass spectrometry

## Abstract

In this work, lipid profile migration from muscle to juice during the tilapia muscle steaming process was revealed by a transactional analysis of data from ultra-high-performance liquid chromatography coupled with Q Exactive (UHPLC-QE) Orbitrap mass spectrometry (MS) and lipidomics. Firstly, the lipids in tilapia muscles and juices at different steaming time points were extracted and examined by UHPLC-QE Orbitrap mass spectrometry. Secondly, a transactional analysis procedure was developed to analyze the data from UHPLC-QE Orbitrap MS and lipidomics. Finally, the corrected lipidomics data and the normalized MS data were used for lipid migration analysis. The results suggested that the transactional analysis procedure was efficient to significantly decrease UHPLC-QE Orbitrap MS workloads and delete the false-positive data (22.4–36.7%) in lipidomics data, which compensated the disadvantages of the current lipidomics method. The lipid changes could be disappearance, full migration into juice, appearance in juice, appearance in muscle, appearance in both muscle and juice, and retention in the muscle. Moreover, the results showed 9 (compared with 52), 5 (compared with 116), and 10 (compared with 178) of lipid class (compared with individual lipid) variables showed significant differences among the different steaming times (0, 10, 30, and 60 min) in all the muscles, juices, and muscle-juice systems, respectively. These results showed significant lipid profile migration from muscle to juice during the tilapia steaming process.

## Introduction

Lipids are basic constituents in foods and play pivotal roles in the nutrition and flavor of foods for energetic, metabolic, and structural activities of human beings^1,2^. Lipids are generally categorized into eight classes: fatty acids, glycerolipids, glycerophospholipids, sphingolipids, sterol lipids, prenol lipids, saccharolipids, and polyketides^3^. It will be beneficial for human diet and health to comprehensively analyze the lipid profile of foods and to understand the lipid profile migration of foods during the production process such as thermal processing.

Lipidomics techniques based on liquid chromatography-mass spectrometry (LC-MS) have been developed to analyze lipid profiles of body fluids and tissues in the last decade^4–7^. In the lipidomics technique, LC-MS data were inputted and analyzed by professional software (e.g., LipidSearch^TM^) with a lipid database for automated identification and relative quantitation of lipid profiles. Ultra-high-performance liquid chromatography coupled with Q Exactive (UHPLC-QE) Orbitrap MS had significant advantages than ordinary LC-MS such as sufficient sensitivity, extremely high resolution, high mass accuracy, and strong power for fragment ion scanning enabling recognition of numerous lipid isomers^8–10^. Recently, lipidomics based on UHPLC-QE Orbitrap MS was applied for lipid profile comparison of goat milk, soymilk, and bovine milk^11^, lipid profile comparison of bee pollen derived from three major nectar plants^12^, and lipid profile identification of marine phospholipids from different resources^13^. In addition, lipidomics technique based on UHPLC/linear trap quadrupole-orbitrap MS was also developed to analyze non-esterified furan fatty acids and fatty acid compositions in dietary shellfish and salmon due to its significant advantages such as high resolution, sufficient sensitivity, and no requirement of derivatization for fatty acids^14^. When the parallel LC-MS results are imported into lipidomics software such as LipidSearch^TM^, only one peak area value is produced for each lipid. Therefore, significantly high LC-MS workloads are needed to produce parallel lipidomics data for subsequent statistical analysis.

Tilapia is an important food resource and is commonly referred to as “aquatic chicken”^13,15^. Due to its excellent advantages such as tender muscle and richness of unsaturated fatty acids, tilapia is the second most important group of global farmed fish after carps^16^. In order to value-addedly utilize tilapia, fresh tilapia is generally processed into frozen fillets in aquatic plants for transportation and sales in the world, especially in Europe, Mexico, USA, etc. Considering that tilapia muscle only contains tiny amounts of lipids and these lipids were rich in unsaturated fatty acids, tilapia muscle is an excellent model to explore the application of lipidomics in foods. UHPLC-electrospray ionization-quadrupole time-of-flight-MS-based lipidomics analyses showed three parts (muscle, head, and viscera) of tilapia showed different lipid profiles and 38 lipids could be potential contributors for differentiation^17^. UHPLC-QE Orbitrap MS-based lipidomics analyses showed three types of thermal processing methods (steaming, boiling, and roasting) had different effects on the lipid profile of tilapia muscles^18^. In addition, UHPLC-QE Orbitrap MS-based lipidomics analyses showed low muscle-to-salt ratios of 3–8 might have stable and similar fatty acid profile changes of tilapia muscles^19^.

Recently, the effect of steaming, an important food processing way in life, on the tilapia muscle has been explored. Steamed tilapia muscle showed different aroma profiles to raw and other thermal processed (boiled, microwaved, and roasted) ones^20^. Steamed tilapia muscle also showed different metabolites profile to raw and other thermal processed (boiled and air fried) ones^21^. Steamed tilapia muscle showed different lipid profiles to raw and other thermal processed (boiled and roasted) ones^18^. However, to the best of our knowledge, though tilapia juice is produced during the steaming process, there are no studies that have presented the aroma or nutritional substances in the tilapia juice and have explored the substance migration from tilapia muscle to juice.

The main objectives of this study were to develop a simple transactional analysis between UHPLC-QE Orbitrap MS data and lipidomics data, and then to analyze the lipid profile migration from tilapia muscle to juice during the steaming process. Firstly, the lipids in tilapia muscles and juices at different steaming time points (0, 10, 30, and 60 min) were extracted and examined by UHPLC-QE Orbitrap mass spectrometry. Secondly, a transactional analysis procedure between lipidomics LipidSearch^TM^ 4.1.3 data and UHPLC-QE Orbitrap MS TraceFinder^TM^ 4.1 data was constructed to obtain the corrected lipidomics data and the normalized MS data for the muscles and juices. Subsequently, the lipid profile migration was analyzed according to the corrected lipidomics data. Finally, the lipid profile migration was statistically analyzed according to normalized MS data by MetaboAnalyst software. All results suggested the transactional analysis procedure was efficient and showed lipid migration profile from tilapia muscle to juice during the steaming process.

## Results

### Transactional analysis of data from UHPLC-QE Orbitrap MS and lipidomics

**Fig. 1:**
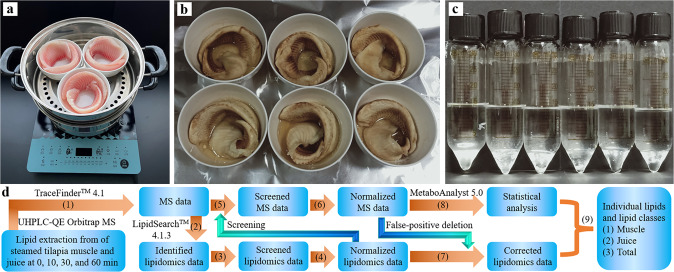
Experimental and analytical process of this work. **a** The bowls with tilapia muscles in a pot on an induction cooker before the steaming process. **b** Tilapia muscles after the steaming process. Steamed tilapia muscles and juices were shown in the bowls. **c** Lipid extraction liquids (about 15 mL of chloroform/methanol). **d** Transactional analysis procedure of data from ultra-high-performance liquid chromatography coupled with Q Exactive (UHPLC-QE) Orbitrap mass spectrometry (MS). MS data indicate the data from Thermo Fisher Scientific TraceFinder^TM^ 4.1 software. Lipidomics data indicate the data from Thermo Fisher Scientific LipidSearch^TM^ 4.1.3 software.

In a typical lipidomics software such as LipidSearch^TM^ 4.1.3 software, several parallel LC-MS results were imported into the software and only one value was exported for each lipid. Therefore, in order to get parallel lipidomics data for subsequent statistical analysis, significantly high LC-MS workloads were needed^18,19^. In this work, tilapia muscles were steamed (Fig. 1a, b), the lipids were extracted (Fig. 1c) and then analyzed by UHPLC-QE Orbitrap MS. Subsequently, a transactional analysis between UHPLC-QE Orbitrap MS data and lipidomics data was developed (Fig. 1d): (1) UHPLC-QE Orbitrap MS data were obtained by TraceFinder 4.1 software; (2) UHPLC-QE Orbitrap MS data were imported into LipidSearch^TM^ 4.1.3 software and compound lipid profiles were identified to obtain the identified lipidomics data; (3) The identified lipidomics data were screened by some key parameters such as MainS/N, IDNum, MainMScore, and MainArea to obtain the screened lipidomics data; (4) In order to correctly compare the compound lipids in muscle and juice, the screened lipidomics data were normalized by dividing the peak area by the initial tilapia muscles to obtain the normalized lipidomics data; (5) The UHPLC-QE Orbitrap MS data were screened by deleting the lipids those were not in the normalized lipidomics data to obtain the screened MS data; (6) In order to correctly compare the compound lipids in muscle and juice, the screened MS data were normalized by dividing the peak area by the initial tilapia muscles to obtain the normalized MS data; (7) The normalized lipidomics data were corrected by deleting the false-positive lipids those were not in the normalized MS data; (8) Statistical analyses of the normalized UHPLC-QE Orbitrap MS data were performed by MetaboAnalyst 5.0 online software; and (9) the individual lipids and lipid classes in muscle, juice, and total system (muscle and juice) were analyzed. The detailed information was shown in the “Methods” section.

As shown in Table 1 and Supplementary Table 1, 16 (compared with 59), 22 (compared with 72), 29 (compared with 79), and 11 (compared with 30) lipids were identified as false positives (compared with corrected ones) in 0, 10, 30, and 60 min, respectively, of tilapia muscles during the steaming process. In addition, 62 (compared with 237), 48 (compared with 214), and 57 (compared with 235) lipids were identified as false positives (compared with corrected ones) in 10, 30, and 60 min, respectively, of tilapia juices during the steaming process. Therefore, according to the relative amounts (22.4–36.7%) of false positives to corrected data, the deletion of false positives was pivotal to obtain corrected lipid profiles in foods.

**Table 1:** The total lipid molecule numbers in muscles and juices during the tilapia muscle steaming processes.

Group	Tilapia muscle												Tilapia soup									Tilapia muscle & soup											
Time	0 min			10 min			30 min			60 min			10 min			30 min			60 min			0 min			10 min			30 min			60 min		
Mode	P	N	M	P	N	M	P	N	M	P	N	M	P	N	M	P	N	M	P	N	M	P	N	M	P	N	M	P	N	M	P	N	M
AcCa	1	0	1	0	0	0	0	0	0	0	0	0	0	0	0	0	0	0	0	0	0	1	0	1	0	0	0	0	0	0	0	0	0
BisMePA	0	0	0	0	0	0	0	0	0	0	0	0	12	0	12	13	0	13	11	0	11	0	0	0	12	0	12	13	0	13	11	0	11
Cer	0	7	7	1	2	3	0	14	14	0	1	1	0	8	8	1	5	6	0	6	6	0	7	7	1	9	9	1	14	15	0	6	6
CL	0	2	2	0	1	1	0	0	0	0	1	1	0	0	0	0	0	0	0	0	0	0	2	2	0	1	1	0	0	0	0	1	1
CmE	0	0	0	0	0	0	0	0	0	0	0	0	1	0	1	0	0	0	0	0	0	0	0	0	1	0	1	0	0	0	0	0	0
DG	2	0	2	0	0	0	0	0	0	0	0	0	27	0	27	26	0	26	34	0	34	2	0	2	27	0	27	26	0	26	34	0	34
dMePE	0	0	0	0	0	0	0	2	2	0	0	0	0	0	0	0	0	0	0	0	0	0	0	0	0	0	0	0	2	2	0	0	0
FA	0	0	0	0	5	5	0	4	4	0	4	4	0	3	3	0	0	0	0	2	2	0	0	0	0	5	5	0	4	4	0	5	5
LPC	0	2	2	3	2	5	11	2	13	8	0	8	5	0	5	5	0	5	6	0	6	0	2	2	7	2	7	13	2	13	9	0	9
LPE	0	0	0	0	0	0	0	6	6	1	0	1	0	0	0	0	0	0	0	0	0	0	0	0	0	0	0	0	6	6	1	0	1
LPG	0	0	0	0	1	1	0	1	1	0	1	1	0	0	0	0	0	0	0	0	0	0	0	0	0	1	1	0	1	1	0	1	1
LPI	0	1	1	0	1	1	0	1	1	0	1	1	0	0	0	0	0	0	0	0	0	0	1	1	0	1	1	0	1	1	0	1	1
LPS	0	1	1	0	1	1	0	0	0	0	0	0	0	0	0	0	0	0	0	0	0	0	1	1	0	1	1	0	0	0	0	0	0
MG	0	0	0	0	0	0	1	0	1	0	0	0	0	0	0	0	0	0	0	0	0	0	0	0	0	0	0	1	0	1	0	0	0
PC	2	5	7	1	1	2	0	3	3	0	1	1	0	13	13	0	3	3	0	8	8	2	5	7	1	14	15	0	5	5	0	9	9
PE	1	13	14	0	3	3	3	10	13	0	2	2	0	3	3	2	0	2	2	1	3	1	13	14	0	6	6	5	10	14	2	3	5
PEt	0	0	0	0	0	0	0	0	0	0	0	0	1	0	1	1	0	1	1	0	1	0	0	0	1	0	1	1	0	1	1	0	1
PG	1	0	1	0	0	0	0	0	0	0	0	0	2	0	2	2	0	2	4	0	4	1	0	1	2	0	2	2	0	2	4	0	4
PI	0	1	1	0	4	4	1	1	2	0	0	0	0	1	1	0	1	1	0	1	1	0	1	1	0	4	4	1	1	2	0	1	1
PS	0	0	0	0	0	0	0	1	1	0	0	0	0	2	2	0	1	1	0	2	2	0	0	0	0	2	2	0	2	2	0	2	2
SM	0	0	0	1	0	1	0	0	0	0	0	0	1	0	1	0	0	0	0	0	0	0	0	0	2	0	2	0	0	0	0	0	0
SPH	0	0	0	0	0	0	0	0	0	0	0	0	2	0	2	3	0	3	2	0	2	0	0	0	2	0	2	3	0	3	2	0	2
TG	19	0	19	45	0	45	17	0	17	10	0	10	156	0	156	150	0	150	155	0	155	19	0	19	168	0	168	158	0	158	158	0	158
WE	1	0	1	0	0	0	1	0	1	0	0	0	0	0	0	0	0	0	0	0	0	1	0	1	0	0	0	1	0	1	0	0	0
Total	27	32	59	51	21	72	34	45	79	19	11	30	207	30	237	203	10	213	215	20	235	27	32	59	224	46	267	225	48	270	222	29	251

P means positive ionization mode. N means negative ionization mode. M means merged positive and negative ionization modes.

This transactional analysis not only could significantly decrease UHPLC-QE Orbitrap MS workloads, but also could delete the false-positive data (Supplementary Table 1) in the lipidomics results to achieve better lipid profile analysis. Therefore, the transactional analytical method may compensate the disadvantages of the current lipidomics analysis method.

### Lipid profile migration analysis according to the corrected lipidomics data

The corrected lipidomics data of individual lipids in muscles, juices, and total system (muscles and juices) during the tilapia muscle steaming process were shown in Supplementary Table 2. All the MS2 data of the lipid ions were checked to confirm the presence of these lipids and typical MS2 data of some uncommon lipid ions were shown in Supplementary Table 3. Three types of significant individual lipid changes could be seen: (1) Disappearance such as acyl carnitin (AcCa) (10:0), ceramide (Cer) (d16:0_22:0), and Cer (d16:1_23:0); (2) Full migration from muscle to juice such as lysophosphatidylcholine (LPC) (18:2) and triglyceride (TG) (18:0_16:0_20:0); (3) Appearance in juice such as bis-methyl phosphatidic acid (BisMePA) (32:1_18:1), BisMePA(32:1_18:2), BisMePA(32:1_18:3), diglyceride (DG) (16:0_18:1), DG(16:0_18:3), DG(16:0_20:4), and DG(16:0_22:4). These results proved that the lipid profile migration from muscle to juice.

Lipid classes in muscles and juices during the tilapia muscle steaming process were shown in Supplementary Table 4. Five types of lipid classes (Cer, LPC, phosphatidylcholine (PC), phosphatidylethanolamine (PE), and TG) were present in both muscle and juice at all the time points during the tilapia steaming process. There were six types of lipid class changes: (1) Disappearance such as AcCa and wax ester (WE); (2) Full migration from muscle to juice such as DG and phosphatidylglycerol (PG); (3) Appearance in juice such as BisMePA, cholesteryl methyl ester (CmE), phosphoethanolamine (PEt), and sphingosine (SPH); (4) Appearance in muscle such as lysophosphatidylethanolamine (LPE), lysophosphatidylglycerol (LPG), dimethylphosphatidylethanolamine (dMePE), and monoglyceride (MG); (5) Appearance in both muscle and juice such as fatty acid (FA), phosphatidylinositol (PI), phosphatidylserine (PS), and sphingomyelin (SM); and (6) Retention in muscle such as cardiolipine (CL), lysophosphatidylinositol (LPI), and lysophosphatidylserine (LPS). These results suggested that the lipid profile migration from muscle to juice.

The corrected lipidomics data of lipid classes in muscles and juices during the tilapia muscle steaming process was shown in Table 2. In the untreated tilapia muscle, TG had a significantly higher amount (E + 14 levels) than other lipid classes, which was consistent with the previous work^18^. After steaming, FA (E + 13 level), LPC (E + 13 level), and TG (E + 14 level) had the top three highest amount among the lipid classes of tilapia muscles. TG (E + 14 level) had also the top highest amount among the lipid classes of tilapia juices. The peak area levels were significantly higher than a typical LC/MS peak area level (E + 9). However, in this work, the detection performance of UHPLC-QE Orbitrap MS was validated by a representative set of quality control samples for retention time-shifts, relative standard deviation (RSD%) of peak area, and mass accuracy^22^, which proved that UHPLC-QE Orbitrap MS could provide significantly higher peak area level (E + 14). Therefore, FA, LPC, and TG had priority to be considered for their nutritional values of steamed tilapia muscles. It should be noted lipidomics software parameters were different for compound lipid analysis and free fatty acid analysis^18,19^. The obtained fatty acid information in the compound lipid analysis might not be comprehensive and accurate. Further comprehensive fatty acids analysis is required to discuss the fatty acid migration from muscle to juice during the tilapia muscle steaming process in the future.

**Table 2:** Corrected lipidomics data of lipid classes in muscles and juices during the tilapia muscle steaming process.

Class	Corrected peak area of lipid in muscle				Corrected peak area of lipid in juice			Total corrected peak area of lipid in muscle and juice			
	0 min	10 min	30 min	60 min	10 min	30 min	60 min	0 min	10 min	30 min	60 min
AcCa	2.80E + 09	ND	ND	ND	ND	ND	ND	2.80E + 09	ND	ND	ND
BisMePA	ND	ND	ND	ND	1.78E + 12	3.06E + 12	2.26E + 12	ND	1.78E + 12	3.06E + 12	2.26E + 12
Cer	5.84E + 11	1.78E + 11	7.35E + 11	1.21E + 10	5.95E + 09	9.76E + 09	1.15E + 10	5.84E + 11	**1.84E** + **11**	**7.45E** + **11**	**2.37E** + **10**
CL	1.70E + 10	3.10E + 10	ND	2.97E + 10	ND	ND	ND	1.70E + 10	3.10E + 10	ND	2.97E + 10
CmE	ND	ND	ND	ND	2.64E + 09	ND	ND	ND	2.64E + 09	ND	ND
DG	1.37E + 11	ND	ND	ND	3.86E + 11	4.12E + 11	6.84E + 11	1.37E + 11	3.86E + 11	4.12E + 11	6.84E + 11
dMePE	ND	ND	5.49E + 11	ND	ND	ND	ND	ND	ND	5.49E + 11	ND
FA	ND	1.59E + 13	1.12E + 13	1.74E + 13	5.20E + 10	ND	3.59E + 10	ND	**1.60E** + **13**	1.12E + 13	**1.74E** + **13**
LPC	1.13E + 11	5.53E + 12	2.39E + 13	2.43E + 13	2.01E + 10	1.92E + 10	2.77E + 10	1.13E + 11	**5.55E** + **12**	**2.39E** + **13**	**2.43E** + **13**
LPE	ND	ND	5.86E + 11	2.30E + 11	ND	ND	ND	ND	ND	5.86E + 11	2.30E + 11
LPG	ND	2.50E + 09	3.07E + 09	1.93E + 09	ND	ND	ND	ND	2.50E + 09	3.07E + 09	1.93E + 09
LPI	1.22E + 10	1.13E + 10	1.62E + 10	2.10E + 10	ND	ND	ND	1.22E + 10	1.13E + 10	1.62E + 10	2.10E + 10
LPS	1.39E + 09	6.05E + 09	ND	2.58E + 09	ND	ND	ND	1.39E + 09	6.05E + 09	ND	2.58E + 09
MG	ND	2.91E + 09	ND	ND	ND	ND	ND	ND	2.91E + 09	ND	ND
PC	8.29E + 12	2.42E + 12	6.65E + 11	1.33E + 11	2.14E + 10	2.21E + 10	2.38E + 10	8.29E + 12	**2.44E** + **12**	**6.87E** + **11**	**1.56E** + **11**
PE	3.72E + 12	3.57E + 11	6.18E + 12	1.17E + 12	1.18E + 09	4.44E + 09	6.82E + 09	3.72E + 12	**3.58E** + **11**	**6.19E** + **12**	**1.17E** + **12**
PEt	ND	ND	ND	ND	3.50E + 09	7.38E + 09	7.06E + 09	ND	3.50E + 09	7.38E + 09	7.06E + 09
PG	1.39E + 12	ND	ND	ND	3.96E + 11	1.68E + 11	7.90E + 11	1.39E + 12	3.96E + 11	1.68E + 11	7.90E + 11
PI	2.50E + 10	5.56E + 11	1.67E + 11	ND	9.76E + 08	1.58E + 09	1.01E + 09	2.50E + 10	**5.57E** + **11**	**1.69E** + **11**	**1.01E** + **09**
PS	ND	ND	6.26E + 11	ND	3.33E + 08	5.82E + 08	1.86E + 09	ND	3.33E + 08	**6.27E** + **11**	1.86E + 09
SM	ND	4.00E + 12	ND	ND	7.30E + 08	ND	ND	ND	**4.00E** + **12**	ND	ND
SPH	ND	ND	ND	ND	2.61E + 10	7.82E + 10	4.28E + 10	ND	2.61E + 10	7.82E + 10	4.28E + 10
TG	1.51E + 14	3.88E + 14	5.95E + 13	1.30E + 13	1.32E + 14	1.59E + 14	1.84E + 14	1.51E + 14	**5.20E** + **14**	**2.19E** + **14**	**1.97E** + **14**
WE	3.48E + 11	ND	5.77E + 11	ND	ND	ND	ND	3.48E + 11	ND	5.77E + 11	ND
Total	1.66E + 14	4.17E + 14	1.05E + 14	5.63E + 13	1.35E + 14	1.63E + 14	1.88E + 14	1.66E + 14	5.52E + 14	2.67E + 14	2.44E + 14

The peak area in bold font were detected in both muscles and juices.

### Lipid profile migration analysis according to the normalized MS data

The normalized MS data contained individual lipids and lipid classes in muscle, juice, and total system (muscle and juice) at different steaming time points (0, 10, 30, and 60 min). Statistical analyses were performed by the partial least squares-discriminate analysis (PLS-DA) module and heat map module in the MetaboAnalyst 5.0 online software. PLS-DA uses multiple linear regression techniques to characterize and classify a data set such as lipids at different steaming time points in this work. The lipids with variable importance in projection (VIP) scores of >1 could be used to differentiate different groups. Heap map visualization could directly provide intuitive visualization of the peak area of the lipids.

**Fig. 2:**
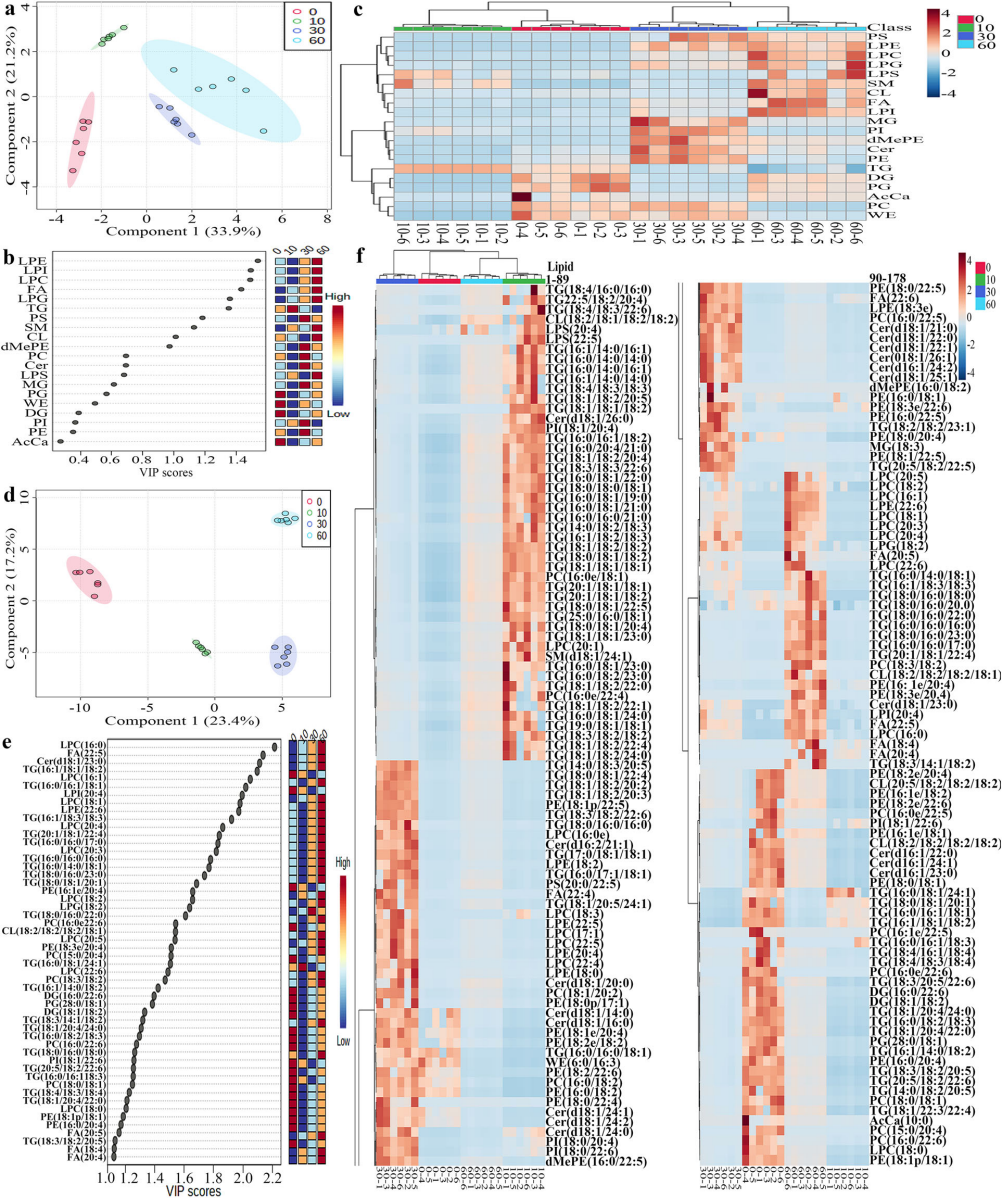
Statistical analyses of lipids in tilapia muscle at different steaming times by Metaboanalyst 5.0 online software according to the normalized MS data. **a** PLS-DA score plot of lipid classes. **b** Variable importance in projection (VIP) scores of lipid classes in PLS-DA. **c** Heat map of lipid classes. **d** PLS-DA score plot of individual lipids. **e** VIP scores of individual lipids in PLS-DA. **f** Heat map of individual lipids. The colored boxes on the right of VIP score images indicate the relative peak area of the corresponding lipid classes. Each colored cell in the heat map corresponds to a peak area value of the lipid classes or individual lipid on the right side. In the colored cells, red color is high and blue color is low.

Statistical analyses of lipids in tilapia muscles were shown in Fig. 2. As shown in the score plot of lipid classes (Fig. 2a), the lipid classes in tilapia muscles at 0, 10, 30, and 60 min were clearly classified according to the first two principal components, and the cumulative contribution rate was 55.1%. As shown in Fig. 2b, nine lipid class variables (LPE, LPI, LPC, FA, LPG, TG, PS, SM, and CL) were accounted to significant contribution (VIP score >1) to tilapia muscles and could be used to differentiate the tilapia muscles at different steaming time points. Heat map visualization **(**Fig. 2c) confirmed tilapia muscles at 0, 10, 30, and 60 min were clearly classified, which was consistent with the analyses of Fig. 2a, b. Further, Fig. 2d showed the individual lipids in tilapia muscles at 0, 10, 30, and 60 min were clearly classified according to the first two principal components, and the cumulative contribution rate was 40.6%. As shown in Fig. 2e, 52 individual lipid variables were accounted to significant contribution (VIP score >1) to tilapia muscles and could be used to differentiate the tilapia muscle at different steaming time points. Heat map visualization (Fig. 2f) confirmed the individual lipids in tilapia muscles at 0, 10, 30, and 60 min were clearly classified, which was consistent with the analyses of Fig. 2d, e.

**Fig. 3:**
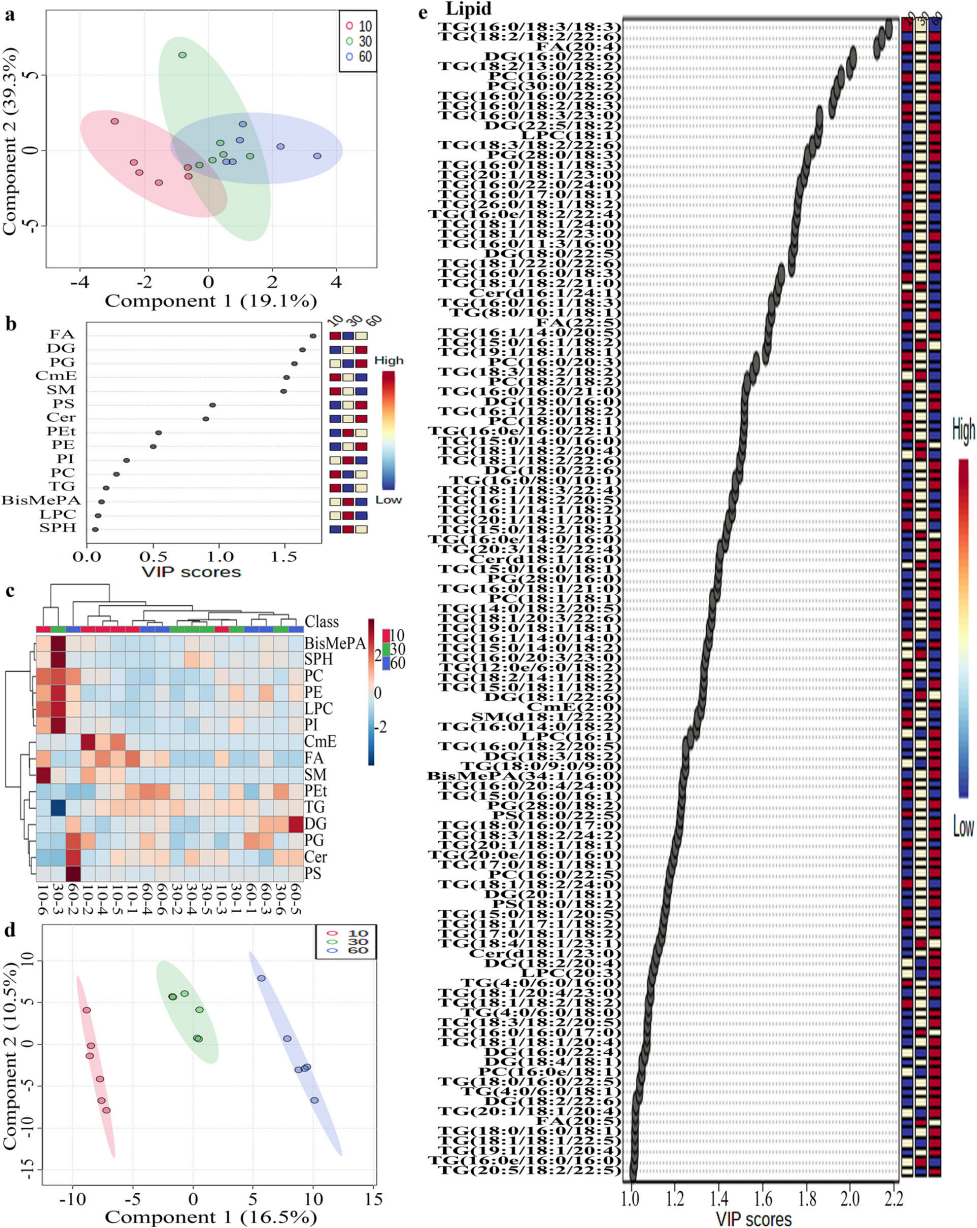
Statistical analyses of lipids in tilapia juice at different steaming times by Metaboanalyst 5.0 online software according to the normalized MS data. **a** PLS-DA score plot of lipid classes. **b** VIP scores of lipid classes in PLS-DA. **c** Heat map of lipid classes. **d** PLS-DA score plot of individual lipids. **e** VIP scores of individual lipids in PLS-DA. The colored boxes on the right of VIP score images indicate the relative peak area of the corresponding lipid classes. Each colored cell in the heat map corresponds to a peak area value of the lipid classes on the right side. In the colored cells, red color is high and blue color is low.

**Fig. 4:**
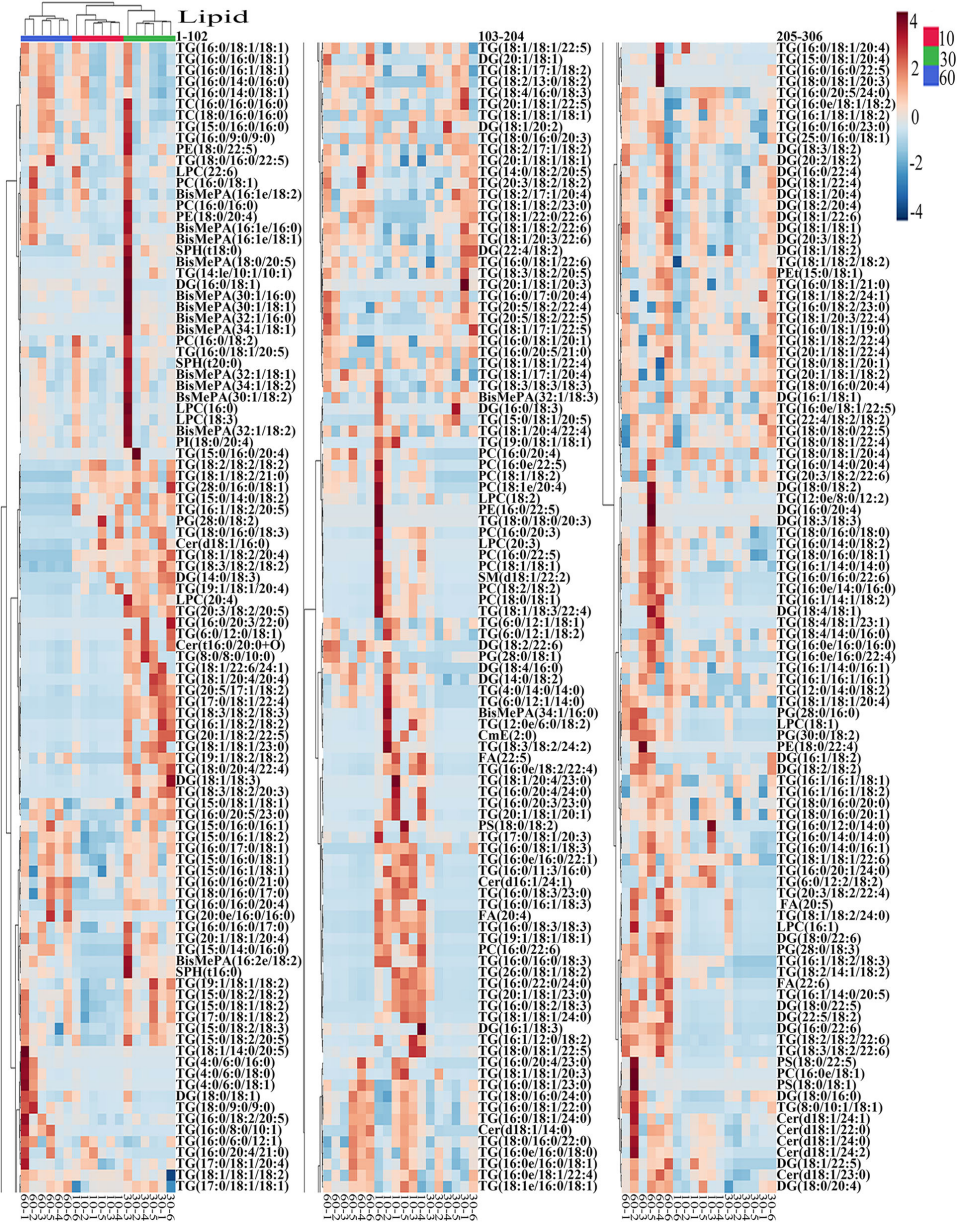
Heap map of individual lipids in tilapia juice at different steaming times by Metaboanalyst 5.0 online software according to the normalized MS data. Each colored cell in the heat map corresponds to a peak area value of the individual lipid on the right side. In the colored cells, red color is high and blue color is low.

Statistical analyses of lipids in tilapia juices were shown in Figs. 3, 4. As shown in the score plot of lipid classes (Fig. 3a), the lipid classes in tilapia juices at 0, 10, 30, and 60 min were not clearly classified. As shown in Fig. 3b, five lipid class variables (FA, DG, PG, CmE, and SM) were accounted to significant contribution (VIP score >1) to tilapia juices and could be used to differentiate the tilapia juices at different steaming time points. Heat map visualization (Fig. 3c) confirmed tilapia muscles at 0, 10, 30, and 60 min were not clearly classified, which was consistent with the analyses of Fig. 3a, b. Further, Fig. 3d showed the individual lipids in tilapia muscles at 0, 10, 30, and 60 min were clearly classified according to the first two principal components, and the cumulative contribution rate was 27.0%. As shown in Fig. 3e, 116 individual lipid variables were accounted to significant contribution (VIP score >1) to tilapia juices and could be used to differentiate the tilapia juices at different steaming time points. Heat map visualization (Fig. 4) confirmed the individual lipids in tilapia juices at 10, 30, and 60 min were clearly classified, which was consistent with the analyses of Fig. 3d, e.

**Fig. 5:**
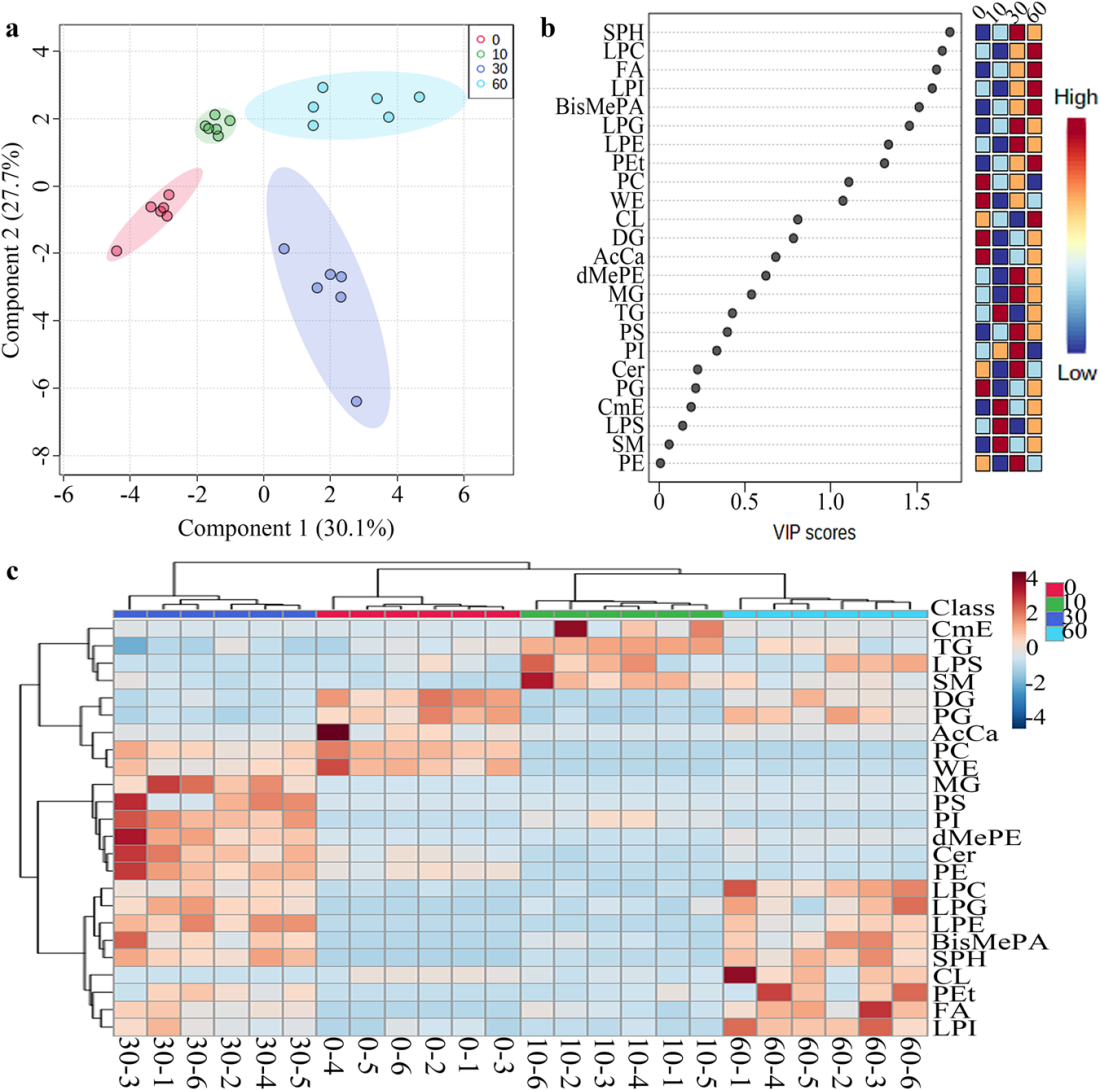
Statistical analyses of the sum lipid class in tilapia muscle and juice at different steaming times by Metaboanalyst 5.0 software according to the normalized MS data. **a** PLS-DA score plot. **b** VIP scores in PLS-DA. **c** Heat map.

**Fig. 6:**
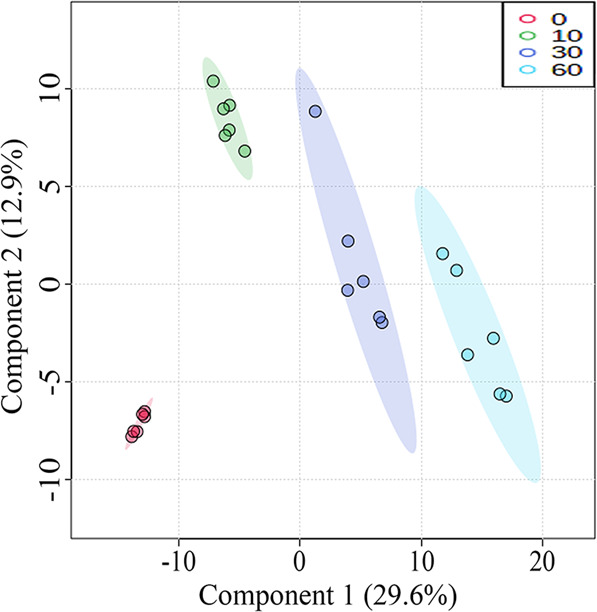
The steaming times for the four groups in different colors are 0, 10, 30, and 60 min. PLS-DA score of the sum individual lipids in tilapia muscle and juice at different steaming times by Metaboanalyst 5.0 software according to the normalized MS data.

**Fig. 7:**
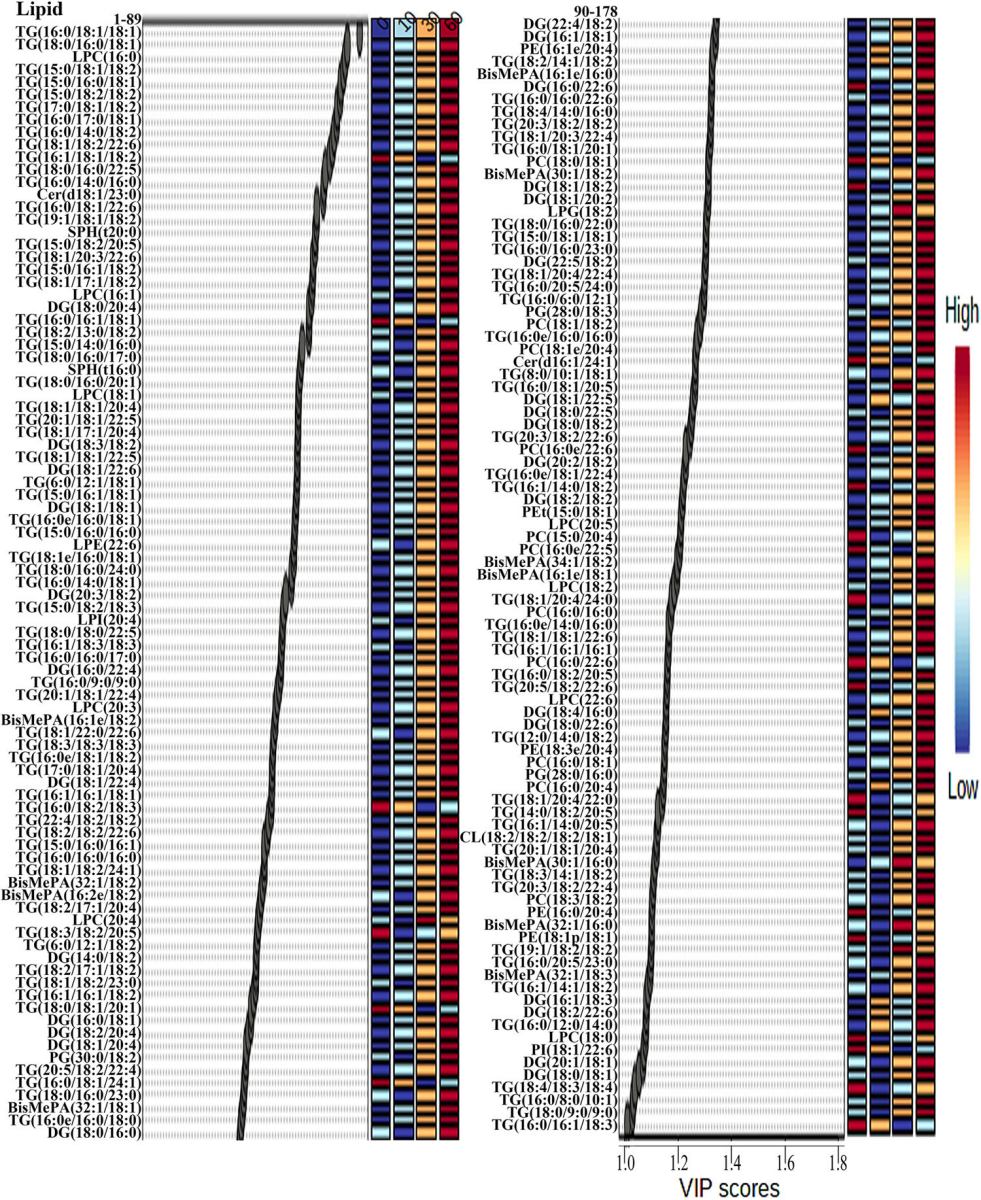
VIP scores in PLS-DA of the sum individual lipids in tilapia muscle and juice at different steaming times by Metaboanalyst 5.0 software according to the normalized MS data. The colored boxes on the right of VIP score images indicate the relative peak area of the corresponding lipid classes. Each colored cell in the heat map corresponds to a peak area value of the lipid classes on the right side. In the colored cells, red color is high and blue color is low.

**Fig. 8:**
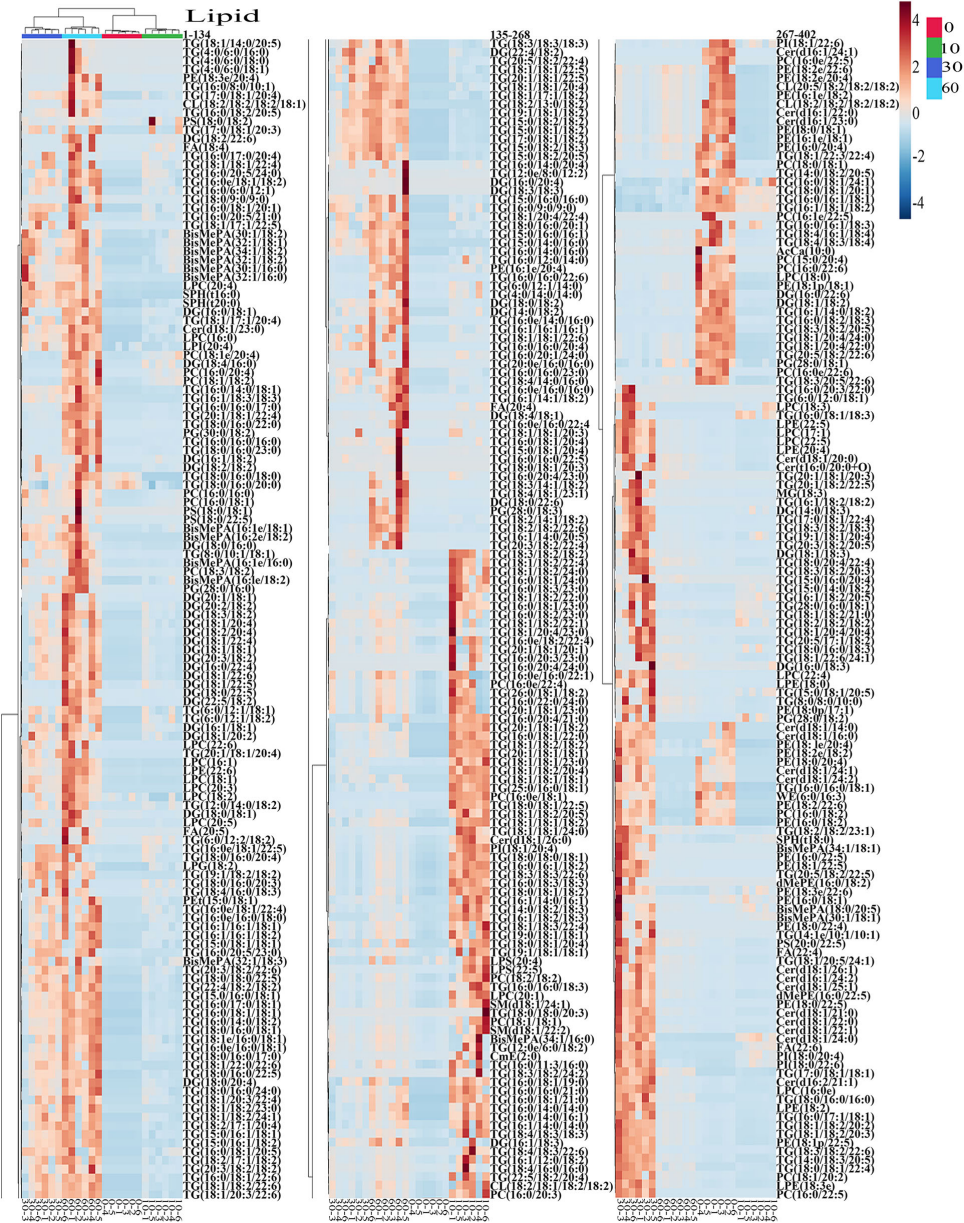
Heap map of the sum individual lipids in tilapia muscle and juice at different steaming times by Metaboanalyst 5.0 software according to the normalized MS data. Each colored cell in the heat map corresponds to a peak area value of the individual lipid on the right side. In the colored cells, red color is high and blue color is low.

Statistical analyses of total lipids in tilapia muscles and juices were shown in Figs. 5–8. As shown in the score plot of lipid classes (Fig. 5a), the total lipid classes in tilapia muscle-juice systems at 0, 10, 30, and 60 min were clearly classified according to the first two principal components, and the cumulative contribution rate was 57.8%. As shown in Fig. 5b, ten lipid class variables (SPH, LPC, FA, LPI, BisMePA, LPG, LPE, Pet, PC, and WE) were accounted to significant contribution (VIP score >1) and could be used to differentiate the total system (muscles and juices) at different steaming time points. It should be noted that these lipid class variables were different from that in tilapia muscle (Fig. 2b) and juice (Fig. 3b). Heat map visualization (Fig. 5c) confirmed total lipid classes in muscle-juice systems at 0, 10, 30, and 60 min were clearly classified, which was consistent with the analyses of Fig. 5a, b. Further, Fig. 6 showed the total individual lipids in muscle-juice systems at 0, 10, 30, and 60 min were clearly classified according to the first two principal components, and the cumulative contribution rate was 42.5%. As shown in Fig. 7, 178 individual lipid variables were accounted to significant contribution (VIP score >1) and could be used to differentiate the muscle-juice systems at different steaming time points. Heat map visualization (Fig. 8) confirmed the total individual lipids in muscle-juice systems at 0, 10, 30, and 60 min were clearly classified, which was consistent with the analyses of Figs. 6, 7.

According to the above analyses, the lipid classes and individual lipids in the muscles, juices, and total muscle-juice systems could be clearly classified except the lipid classes in juices during the tilapia muscle steaming process. Moreover, 9 (compared with 52), 5 (compared with 116), and 10 (compared with 178) of lipid class (compared with individual lipid) variables could be used to differentiate the lipid profiles at different steaming times (0, 10, 30, and 60 min) in the muscles, juices, and total muscle-juice systems, respectively.

## Discussion

LC-MS-based lipidomics has already been developed to analyze lipid profiles of body fluids and tissues in the last decade^4^. Recently, it was applied to compare lipid profiles of goat milk, soymilk, and bovine milk^11^, and lipid profiles of bee pollen derived from three major nectar plants^12^. In addition, it was also applied to identify lipid profiles of marine phospholipids^13^. This technique was also used to compare the lipid profile of tilapia muscles after three types of thermal processing methods^18^ and analyze the effect of dry salting on the long-chain free fatty acid profile of tilapia muscles^19^. However, significantly high LC-MS workloads were needed to produce parallel lipidomics data for subsequent statistical analysis. In addition, these works generally focused on the application of lipidomics and neglected the deletion of false positives after lipidomics software identification.

Steaming is a typical traditional thermal processing method to cook foods using steam, which is generated by boiling water continuously^23^. Several studies suggested that steaming is the most efficient thermal processing method to maximize the nutritional value and sensory characteristics of foods^24,25^. In addition, several studies have already explored the effects of steaming on flavor substances and protein degradation^26^, antioxidant activity^23^, contaminants^27^, and lipid profiles^18^ in foods. However, to the best of our knowledge, no studies have already reported on the substance migration from foods to juices during the steaming process.

In this work, we developed a transactional analysis procedure for data from UHPLC-QE Orbitrap MS and lipidomics to significantly decrease UHPLC-QE Orbitrap MS workloads and delete the false-positive data in lipidomics (Fig. 1d). According to our practice, no more UHPLC-QE Orbitrap MS workloads were needed to obtain parallel data for statistical analysis (Figs. 2–8). In addition, as shown in Table 1 and Supplementary Table 1, the ratios of false positives to corrected lipids were between 22.4 and 36.7%. Therefore, the transactional analysis procedure could efficiently achieve believable lipid profile analysis. Though lipidomics is a powerful platform for lipid research^5^, our work implied that we should be careful to analyze the lipidomics data and consider the potential false positives in the future.

After the transactional analysis, the corrected lipidomics data could be applied to analyze the lipid profile migration. As shown in Table 2, FA, LPC, and TG have priority to be considered for their nutritional values of steamed tilapia muscles. Our previous work also suggested LPC and TG were significantly higher than other compound lipids^18^. In addition, the normalized MS data could be applied for statistical analyses of lipid profiles at different steaming time points by MetaboAnalyst 5.0 online software. As shown in Figs. 2–8, the lipid classes and individual lipids in the muscles, juices, and total muscle-juice systems could be clearly classified except the lipid classes in juices during the tilapia muscle steaming process. Therefore, lipids migration profiles from muscle to juice during the tilapia muscle steaming process were shown in this work.

Lipid classes in muscles and juices during the tilapia muscle steaming process were shown in Supplementary Table 4. Five types of lipid classes (Cer, LPC, PC, PE, and TG) were present in both muscle and juice at all time points during the tilapia steaming process. These lipids are amphipathic lipids and may form liposomes in water^28–32^. Therefore, these lipid classes might migrate from muscle to juice and form liposomes in the juice. In addition, there were six types of lipid class changes: (1) Disappearance such as AcCa(10:0) and WE(6:0_16:3). These two were only detected in raw tilapia muscles (Table 1 and Supplementary Table 2). They might be destroyed due to heat-induced hydrolysis. (2) Full migration from muscle to juice such as DG and PG. DG and PG are amphipathic lipids and may form liposomes in water^33,34^. Therefore, these lipid classes might completely migrate from muscle to juice and form liposomes in the juice. (3) Appearance in juice such as BisMePA, CmE, PEt, and SPH. BisMePA might be formed due to heat-induced hydrolysis of the lipids in muscles such as PC and PE. CmE(2:0) was only be detected in the tilapia juice at 10 min and might be formed due to heat-induced hydrolysis of cholesterol-related chemicals. PEt(15:0_18:1) was detected in the tilapia juice at 10, 30, and 60 min. It might be formed due to heat-induced hydrolysis of the lipids in muscles such as PE. SPH(t16:0, t18:0, and t20:0) might be formed due to heat-induced hydrolysis of the lipids in muscles such as sphingomyelin. (4) Appearance in muscles such as LPE, LPG, dMePE, and MG. They might be formed due to heat-induced hydrolysis of PE, PG, PE, and DG/TG, respectively. (5) Appearance in both muscle and juice such as FA, PI, PS, and SM. FA might be formed due to heat-induced hydrolysis of DG and TG. PI, PS, and SM might be formed due to heat-induced hydrolysis of glycosylphosphatidylserine, glycosylphosphatidylinositol, and glycosylSphingomyelin, respectively. (6) Retention in muscles such as CL, LPI, and LPS. These results suggested that the lipid profile migration from muscle to juice. It should be noted our study mainly used the untargeted lipidomics technique to analyze the lipid profile migration, which could not provide accurate lipid origins and products during the steaming process^35^. This weakness could be solved by using targeted lipidomics in the future^36^.

In summary, a transactional analysis procedure for data from UHPLC-QE Orbitrap MS and lipidomics was developed in this work, which could significantly decrease UHPLC-QE Orbitrap MS workloads and delete the false-positive data in lipidomics. Further, the lipid profile migration from muscle to juice during the tilapia muscle steaming process was studied. The results showed lipids could differently migrate from muscle to juice during the tilapia muscle steaming process and the lipid profiles at different steaming time points were significantly different in muscles, juices, and muscle-juice systems. The migration of various molecular species was important for the lipid profile migration research during the steaming process and the adding of internal standards (at least one for each lipid class) might interrupt the lipid measurement. Therefore, we did not use internal standards to analyze the relative lipid amounts as mg/g of tilapia muscle, which might be the major shortcoming of this work. However, this work could provide an efficient method to compensate the disadvantages of the current lipidomics analysis method. Moreover, this work could provide useful information to understand the effect of thermal processing methods on the lipid profiles of foods.

## Methods

### Reagents

All chemicals for lipid extraction and examination were of chromatographic grade. Methanol, acetonitrile, and ammonium acetate were purchased from Fisher Chemical (Geel, Belgium). Chloroform was purchased from Anaqua Chemicals (Houston, Texas, USA). Isopropanol was purchased from Tedia Company. Inc. (Fairfield, Ohio, USA).

### Steaming process of tilapia muscles

Frozen Genetic Improvement of Farmed Tilapia fillets (180–200 g for each) were bought from Hainan Xiangtai Fishery Co., Ltd. (Chengmai County, Hainan Province, China). The frozen tilapia fillets were shipped to our laboratory, stored at −20 °C in our laboratory, and were not used after two months. Frozen tilapia fillets were naturally thawed in ultrapure water for 30–60 min at room temperature. Then, the tilapia fillets were dried, weighed, and cut into two halves. Each tilapia fillet was placed in a dried and clean bowl with a bowl edge diameter of 12.5 cm (Fig. 1a). Three liters of ultrapure water was put into a pot on an induction cooker (C21-WT2118, Guangdong Media, China) and then the ultrapure water was boiled at an operational power of 2100 kW, which was generally used to quickly boil water prior to steaming in life. Subsequently, the bowls with tilapia fillets were put into the pot for steaming food. Immediately, the tilapia fillets were steamed for 10, 30, and 60 min with an operational power of 1600 kW, which was generally used to steam foods in life. At the designated time points, the induction cooker was turned off and the bowls were moved onto silver paper on the experimental table (Fig. 1b). All the samples were randomly selected and prepared in hexaplicates and the data were expressed as average value ± standard deviation.

### Lipid extraction from tilapia muscles

The lipid extraction from tilapia muscles was performed according to Folch and Bligh methods^37,38^ with minor modification. Briefly, at the designated time points, the tilapia muscles were cooled at room temperature and weighed. Tilapia muscles with or without steaming were chopped using a Model QSJ-A01P6 chop machine (Guangdong Bear Electric Co. Ltd., China) for 3–5 min. Subsequently, 5 mL chloroform/methanol (2:1, v/v) was added into 1.0 g of tilapia muscle paste. The mixture was vortexed by a vortex oscillator (QL-901, Kylin-Bell Lab Instruments Co., Ltd., Haimen City, Jiangsu Province, China) for one min at room temperature, shaken on a constant temperature shaker (THZ-C, Taicang Haocheng Laboratory Instrument Manufacture Co. Ltd., Taicang City, Jiangsu Province, China) operated at 200 rpm at room temperature for 10 min, and then centrifuged at 1274 × g (H1850R, Changsha Xiangyi Centrifuge Instrument Co., Ltd., Hunan Province, China) for 15 min at 4 °C. The organic phase was transferred to a new 30 mL of glass tube. The chloroform/methanol extraction process was repeated twice for the residue tilapia paste. In the last extraction process, the centrifugation speed was adjusted to 15,285 × *g*. The organic phases were added into the new 30 mL of glass tube (Fig. 1c). The collected 15 mL of organic solvents were dried for 15 min at low pressure at 35 °C by a rotary evaporator (RE52AA, Yarong Corporation, Shanghai, China) to obtain stable volume (about 300 µL), which could not be dried any more. Finally, the samples were freeze-dried for 12 h.

### Lipid extraction from tilapia juices

At different designated time points, tilapia juices were collected and the volumes were measured. Then, 5 mL of chloroform/methanol (2:1, v/v) was added into 1.0 mL of tilapia juice. The mixture was vortexed by a vortex oscillator for one min at room temperature, shaken on a constant temperature shaker operated at 200 rpm at room temperature for 10 min, and then centrifuged at 1274 × *g* for 15 min at 4 °C. The organic phase was transferred to a new 30 mL of glass tube. The chloroform/methanol extraction process was repeated twice for the residue tilapia juice phase. In the last extraction process, the centrifugation speed was adjusted to 15,285 × *g*. The organic phases were collected and were concentrated for 15 min at low pressure at 35 °C by a rotary evaporator to obtain stable volume (about 300 µL), which could not be dried any more. Finally, the samples were freeze-dried for 12 h.

### Instruments and methods

The compound lipids were examined by a UHPLC-QE Orbitrap MS (Thermo Fisher Scientific, CA, USA) with a heated electrospray ionization probe according to a previous work^18^. Briefly, 5.0 mL of chloroform/methanol (2:1, v/v) was added into the sample glass tube, and was vortexed (Eyela Cute Mixer CM-1000, Tokyo Rikakikai Co., Tokyo, Japan) for ten min at room temperature. The solution was filtered through a 0.22 μm pore size nylon syringe filter (Thermo Fisher Scientific, San Jose, CA, USA)^39^. Then, 2 μL of the filtered solution was injected into the UHPLC-QE Orbitrap MS system with an ACQUITY UPLC HSS T3 100 × 2.1 mm 1.8 μm column (Waters, USA).

The experiments were performed at a spray voltage of 3000 V, a heater temperature of 300 °C, a capillary temperature of 320 °C, a sheath gas flow rate of 35 Arb, an auxiliary gas flow rate of 10 Arb, a column chamber of 45 °C, and an instrument default sample tray temperature of 10 °C to maintain the sample biological activity and to minimize the solvent evaporation. Lipid sample was eluted by a binary solvent system at a flow speed of 300 μL/min^11^: (i) mobile phase A: acetonitrile: water (60:40, v/v) and 10 mM ammonium acetate, and (ii) mobile phase B: isopropanol: acetonitrile (90:10, v/v) and 10 mM ammonium acetate. The elution process was begun with an initial flow phase equilibrium in four min, a linear gradient increase from 37% mobile phase B to 98% mobile phase B in 20 min, 98% mobile phase B in 6 min, a linear gradient decrease of mobile phase B from 98 to 37% in 3 min, and a re-equilibration in 4 min with 37% mobile phase B. The performance of the MS detection was validated by monitoring a representative set of quality control samples for retention time-shifts, RSD% of peak area and mass accuracy^22^. The quality control samples were prepared by pooling equal volumes of all the samples together. Data were acquired with dependent MS/MS acquisition in positive ionization mode (*m*/*z* 240–2000) and negative ionization mode (*m*/*z* 200–2000). Full scan spectra and fragment spectra used a resolution of 70,000 and 17,500, respectively. Representative UHPLC-Extractive Orbitrap MS base peak intensity chromatograms acquired in positive and negative ionization modes of the quality control sample and tilapia muscle at 0 min were shown in Supplementary Figs. 1–4, which confirmed that MS instrument was not saturated with the lipids.

### Transactional analysis of data from UHPLC-QE Orbitrap MS and lipidomics

A transactional analysis procedure was constructed to analyze the data from UHPLC-QE Orbitrap MS and lipidomics (Fig. 1d). The detailed procedure was introduced in this section.

### Identification and screening of lipidomics data

The Lipidsearch^TM^ 4.1.3 software (Thermo Fisher Scientific, CA, USA) was applied to analyze UHPLC-QE Orbitrap MS data for compound lipid identification in the tilapia muscles and juices^18^. The identified lipidomics data (compound lipid tables) were obtained in both positive and negative ionization modes. In these tables, the lows with MainS/N < 5, IDNum < 3, MainMScore < 5, or MainArea < 1 × 10^5^ were deleted. If the compound lipid had multiple rows, the rows with lower MainArea, lower IDnum, or lower MainS/N were deleted and only one row was left for each compound lipid. Then, the data from positive and negative ionization modes were merged into one table and the rows with lower MainArea were deleted and only one row was left for each compound lipid. Therefore, the compound lipids were screened in both steamed tilapia muscles and juices to obtain screened lipidomics data.

### Normalization of screened lipidomics data

In order to correctly compare the compound lipids in muscle and juice, the peak area of each compound lipid in tilapia muscle and juice were normalized by dividing the peak area by the initial tilapia muscles:

1 $$P_{mn} = \frac{{{{{\mathrm{P}}}}_m \times 5\,{{{\mathrm{mL}}}} \times M_{ms}}}{{2\,\upmu {{{\mathrm{L}}}} \times 0.001\,{\rm{mL}}/\upmu L \times 1.0\;g \times M_{m0}}}$$

2 $$P_{sn} = \frac{{{{{\mathrm{P}}}}_s \times 5\,{{{\mathrm{mL}}}} \times V_{ss}}}{{2\,\upmu {{{\mathrm{L}}}} \times 0.001\,\rm{mL}/\upmu L \times 1.0\;mL \times M_{m0}}}$$

where $P_{mn}$ and $P_{sn}$ were normalized peak area of each compound lipid in tilapia muscle and juice, respectively. $P_{mn}$ and $P_{sn}$ were analyzed peak area of each compound by LipidSearch^TM^ 4.1.3 software in tilapia muscle and juice, respectively. $M_{ms}$ and $V_{ss}$ were the mass of tilapia muscle and the volume of tilapia juice, respectively, at the designated time points after steaming. $M_{m0}$ was the mass of initial fresh tilapia muscle. The obtained tables were the normalized lipidomics data.

### Screening of UHPLC-QE Orbitrap MS data

In the UHPLC-QE Orbitrap MS data, the compound lipids were deleted if they were not in the normalized lipidomics data. Then, the data from positive and negative ionization modes were merged into one table. Subsequently, these tables were screened according to the screening standards in the identification and screening processes of compound lipid profile by LipidSearch^TM^ 4.1.3 software (section of normalization of screened lipidomics data). Briefly, the lows with m-Score < 5 or MainArea < 1 × 10^5^ were deleted. If the compound lipid had multiple rows with different ionization modes, the rows with lower MainArea or lower m-Score were deleted. If the compound lipid had multiple rows with different quantified ions, the MainAreas were added together to replace the row with large MainAreas and other rows were deleted. Subsequently, the IDNum of each compound lipid at different steaming time was summarized. The compound lipids with IDNum < 3 were deleted. Therefore, the compound lipids were screened in both steamed tilapia muscles and juices to obtain the screened MS data.

### Normalization of screened UHPLC-QE Orbitrap MS data

In order to correctly compare the compound lipids in muscle and juice, the peak area of each compound lipid in screened UHPLC-QE Orbitrap MS data were normalized according to Eqs. (1) and (2) in the section of normalization of screened lipidomics data. The total normalized peak area of each compound lipid in the steaming process were calculated by adding the normalized peak area of each compound lipid in tilapia muscle and juice together. Then, the average value and standard deviation were calculated for each compound lipid.

### Statistical analysis of normalized MS data

The normalized peak area of each lipid category in the steaming process was calculated by adding the total normalized peak area of every compound lipid in this category together. The parallel normalized UHPLC-QE Orbitrap MS data were imported in MetaboAnalyst 5.0 online software^40,41^. The data were analyzed by PLS-DA, VIP scores, and heat map visualization. The statistics were performed by one-way ANOVA with adjusted *p* value of <0.05 and post hoc analyses of Fisher’s LSD.

### Correcting of lipidomics data

For both steamed tilapia muscles and juices, the lipidomics data after normalization (section of normalization of screened lipidomics data) were corrected by deleting the false-positive lipids those were not in the normalized MS data. After deletion of the false-positive data, the compound lipids in lipidomics tables were categorized and the normalized peak area of each compound lipid category were calculated by adding the lipid peak area in this lipid category together. The total normalized peak area of each compound lipid in the steaming process were calculated by adding the normalized peak area of each compound lipid in tilapia muscle and juice together. The total normalized peak area of each lipid category in the steaming process was calculated by adding the total normalized peak area of every compound lipid in this category together.

### Reporting summary

Further information on research design is available in the Nature Research Reporting Summary linked to this article.

## Supplementary information

Supplementary Information

Reporting Summary

## Data Availability

The authors declare that all data supporting the findings of this study are available in the paper and supplementary information.
