# Supplementary material for: Lipid profile migration during the tilapia muscle steaming process revealed by a transactional analysis between MS data and lipidomics data
Source: NPJ Sci Food. 2021 Nov 15;5:30. doi: 10.1038/s41538-021-00115-1 (PMC8593017; doi:10.1038/s41538-021-00115-1)
Supplement: Supplementary file 1 — Supplementary Information [file 41538_2021_115_MOESM1_ESM.pdf]

Lipid profile migration during the tilapia muscle steaming process revealed by a transactional analysis between MS data and lipidomics data

Rui Sun,[1] Tingting Wu,[1] Hao Guo,[2] Jiamin Xu,[1] Jiahui Chen,[1] Ningping Tao,[1] Xichang Wang,[1] Jian Zhong.[1,3,*]

[1]National R & D Branch Center for Freshwater Aquatic Products Processing Technology (Shanghai), Integrated Scientific Research Base on Comprehensive Utilization Technology for By-Products of Aquatic Product Processing, Ministry of Agriculture and Rural Affairs of the People's Republic of China, Shanghai Engineering Research Center of Aquatic-Product Processing and Preservation, College of Food Science & Technology, Shanghai Ocean University, Shanghai 201306, China.

[2]Chongqing Institute of Forensic Science, Chongqing 400021, China.

[3]Collaborative Innovation Center of Seafood Deep Processing, Dalian Polytechnic University, Dalian 116034, Liaoning Province, China

[*]Corresponding authors. E-mail: jzhong@shou.edu.cn (J. Z.). Phone: 0086-21-61900363. Fax: 0086-21-61900365

Abbreviated running title: Lipid profile migration from tilapia muscle to juice

Supplementary Table 1. False positive lipids in the Lipidomics data

Group	0 min	10 min	30 min	60 min
Muscle	Cer(d16:2_21:1)	Hex1Cer(d40:1)	Cer(d36:0)	FA(22:6)
	Cer(d34:0)	OAHFA(40:4)	FA(18:4)	OAHFA(40:7)
	PC(32:1)	OAHFA(40:7)	LPE(18:3e)	OAHFA(42:10)
	PC(32:2)	PC(14:0_22:1)	LPE(22:6)	PC(20:1_18:1)
	PC(35:2)	PC(14:0_22:2)	OAHFA(34:2)	PC(37:3)
	PC(36:3)	PC(20:1_18:1)	OAHFA(36:1)	PC(37:6)
	PC(38:4)	PC(34:1e)	OAHFA(36:3)	PE(18:2e)
	PC(38:6)	PC(38:1)	OAHFA(38:4)	PE(20:4e)
	PC(40:5)	PC(38:2)	OAHFA(40:7)	PEt(34:6e)
	PC(40:9e)	PC(38:3)	OAHFA(42:10)	SM(d42:1)
	PC(42:7)	PEt(34:6e)	OAHFA(44:11)	TG(12:0_18:3_18:3)
	SM(d34:1)	SM(d32:1)	OAHFA(44:9)	
	SM(d36:4)	SM(d38:1)	OAHFA(46:10)	
	SM(d41:1)	SM(d41:1)	PC(19:5e)	
	SM(d42:1)	SM(d41:2)	PC(20:1_18:1)	
	TG(20:5_14:1_18:2)	SM(d42:1)	PC(37:3)	
		SM(d42:2)	PC(39:5)	
		SM(d44:5)	PC(40:4)	
		TG(16:1_12:0_18:2)	PC(40:6)	
		TG(16:1_18:1_18:1)	PC(42:6)	
		TG(18:0_16:0_21:0)	PE(18:0e)	
		TG(18:0_18:0_20:4)	PE(18:1e)	
			PE(22:5e)	
			PEt(34:6e)	
			SM(d32:1)	
			SM(d34:1)	
			SM(d40:1)	
			SM(d42:1)	
			SM(d42:2)	
Juice	NA	BisMePA(16:1e_16:1)	Cer(d32:1+O)	Cer(d16:1_24:1)
		Cer(d32:0)	DG(14:0_18:2)	Cer(d32:0)
		Cer(d32:1+O)	PC(20:3e_16:0)	Cer(d32:1+O)
		Cer(d34:0)	PC(20:3e_18:2)	Cer(d34:0)
		Cer(t34:0+O)	PC(20:4e_16:0)	OAHFA(36:1)
		PC(18:2e_20:4)	PC(30:0)	OAHFA(38:4)
		PC(20:3e_16:0)	PC(31:0)	OAHFA(38:6)
		PC(20:3e_18:2)	PC(32:0)	PC(18:2e_20:4)
		PC(20:3e_20:3)	PC(32:0e)	PC(20:3e_18:2)
		PC(20:4e_16:0)	PC(32:1)	PC(20:4e_16:0)
		PC(30:0)	PC(32:2)	PC(30:0)
		PC(31:0)	PC(34:1)	PC(32:0)
		PC(32:0)	PC(34:1e)	PC(32:1)
		PC(32:1)	PC(34:2)	PC(34:1)
		PC(32:2)	PC(34:3)	PC(34:2)
		PC(34:1)	PC(36:1)	PC(36:1)
		PC(34:1e)	PC(36:4)	PC(36:4)
		PC(34:2)	PC(38:5)	PC(38:5)
		PC(34:2e)	PC(38:6)	PC(38:6)
		PC(36:1)	PC(40:6)	PC(40:6)
		PC(36:2)	PC(40:7)	PC(40:7e)
		PC(36:3)	PC(40:7e)	PE(16:0p_22:6)
		PC(36:4)	PC(40:8)	PE(20:3_20:3)
		PC(38:5)	SM(d32:1)	PG(18:3e)
		PC(38:6)	SM(d34:1)	PG(26:0_18:2)
		PC(40:5)	SM(d40:1)	SM(d32:1)
		PC(40:7e)	SM(d40:2)	SM(d32:1)
		PG(18:3e)	SM(d42:2)	SM(d34:1)
		PG(28:0_16:0)	SM(d42:3)	SM(d36:1)
		PG(28:0_18:3)	TG(15:0_18:1_20:4)	SM(d38:1)
		SM(d32:1)	TG(16:0_20:1_24:0)	SM(d40:1)
		SM(d34:0)	TG(16:1e_16:0_18:1)	SM(d40:2)
		SM(d34:1)	TG(18:0_18:0_20:3)	SM(d41:1)
		SM(d36:1)	TG(18:0e_18:1_18:2)	SM(d42:2)
		SM(d38:1)	TG(18:1_14:0_20:5)	SM(d42:3)
		SM(d40:1)	TG(18:1_18:1_22:0)	TG(15:0_18:1_20:5)
		SM(d40:2)	TG(18:1_18:1_24:0)	TG(16:0_12:0_20:4)
		SM(d42:2)	TG(18:1_18:2_22:1)	TG(16:0_22:0_22:0)
		SM(d42:3)	TG(18:1_18:2_24:0)	TG(16:1e_16:0_18:1)
		TG(15:0_14:0_16:1)	TG(18:1_18:3_22:0)	TG(18:0_18:0_20:3)
		TG(16:0_20:5_23:0)	TG(19:0_18:1_18:1)	TG(18:0_20:1_22:5)
		TG(16:0_22:0_22:0)	TG(19:1_18:1_18:1)	TG(18:0_20:4_22:4)
		TG(16:1e_16:0_18:1)	TG(19:1_18:1_20:5)	TG(18:0e_18:1_18:2)
		TG(18:0_20:4_22:4)	TG(20:1_18:1_20:1)	TG(18:1_18:1_22:0)
		TG(18:0_9:0_9:0)	TG(20:3_18:2_22:4)	TG(18:1_18:1_23:0)
		TG(18:0e_18:1_18:2)	TG(25:0_16:0_16:0)	TG(18:1_18:1_24:0)

	TG(18:1_14:0_20:5)	TG(27:0_16:0_18:1)	TG(18:1_18:2_21:0)
	TG(18:1_18:1_22:0)	TG(8:0_8:0_8:0)	TG(18:1_18:2_22:1)
	TG(18:1_18:2_22:1)		TG(18:1_18:2_24:2)
	TG(18:1_18:2_23:0)		TG(19:0_18:1_18:1)
	TG(18:1_18:2_24:0)		TG(19:1_18:1_18:1)
	TG(19:1_18:1_20:5)		TG(20:1_18:1_20:1)
	TG(20:0e_16:0_16:0)		TG(20:1_18:2_22:5)
	TG(20:1_18:1_20:3)		TG(25:0_16:0_16:0)
	TG(20:1_18:2_22:5)		TG(27:0_16:0_18:1)
	TG(25:0_16:0_16:0)		TG(28:1_16:0_18:1)
	TG(25:0_16:0_18:0)		TG(6:0_12:0_18:1)
	TG(27:0_16:0_18:1)		
	TG(28:1_16:0_18:1)		
	TG(36:3)		
	TG(6:0_12:0_18:1)		
	TG(9:0_18:1_18:3)		

Supplementary Table 2. Corrected lipidomics data of individual lipids in muscles and juices during the tilapia muscle steaming process.

Lipid	Corrected peak area of lipid in muscle				Corrected peak area of lipid in juice			Total corrected peak area of lipid in muscle and juice			
	0 min	10 min	30 min	60 min	10 min	30 min	60 min	0 min	10 min	30 min	60 min
AcCa(10:0)	2.80E+09	ND	ND	ND	ND	ND	ND	2.80E+09	ND	ND	ND
BisMePA(16:1e_16:0)	ND	ND	ND	ND	1.50E+09	5.12E+09	1.25E+10	ND	1.50E+09	5.12E+09	1.25E+10
BisMePA(16:1e_18:1)	ND	ND	ND	ND	2.19E+09	4.57E+09	4.60E+09	ND	2.19E+09	4.57E+09	4.60E+09
BisMePA(16:1e_18:2)	ND	ND	ND	ND	3.05E+09	5.73E+09	4.60E+09	ND	3.05E+09	5.73E+09	4.60E+09
BisMePA(16:2e_18:2)	ND	ND	ND	ND	ND	2.68E+09	2.43E+09	ND	ND	2.68E+09	2.43E+09
BisMePA(18:0_20:5)	ND	ND	ND	ND	2.25E+09	ND	ND	ND	2.25E+09	ND	ND
BisMePA(30:1_16:0)	ND	ND	ND	ND	3.58E+10	4.72E+10	4.76E+10	ND	3.58E+10	4.72E+10	4.76E+10
BisMePA(30:1_18:1)	ND	ND	ND	ND	1.21E+11	2.15E+11	ND	ND	1.21E+11	2.15E+11	ND
BisMePA(30:1_18:2)	ND	ND	ND	ND	6.44E+10	1.04E+11	8.40E+10	ND	6.44E+10	1.04E+11	8.40E+10
BisMePA(32:1_16:0)	ND	ND	ND	ND	ND	1.69E+11	1.62E+11	ND	ND	1.69E+11	1.62E+11
BisMePA(32:1_18:1)	ND	ND	ND	ND	3.90E+11	6.09E+11	4.50E+11	ND	3.90E+11	6.09E+11	4.50E+11
BisMePA(32:1_18:2)	ND	ND	ND	ND	5.01E+11	8.06E+11	6.18E+11	ND	5.01E+11	8.06E+11	6.18E+11
BisMePA(32:1_18:3)	ND	ND	ND	ND	3.08E+11	4.79E+11	5.49E+11	ND	3.08E+11	4.79E+11	5.49E+11
BisMePA(34:1_16:0)	ND	ND	ND	ND	5.36E+10	ND	ND	ND	5.36E+10	ND	ND
BisMePA(34:1_18:1)	ND	ND	ND	ND	ND	1.54E+11	ND	ND	ND	1.54E+11	ND
BisMePA(34:1_18:2)	ND	ND	ND	ND	3.01E+11	4.63E+11	3.20E+11	ND	3.01E+11	4.63E+11	3.20E+11
Cer(d16:1_22:0)	3.10E+10	ND	ND	ND	ND	ND	ND	3.10E+10	ND	ND	ND
Cer(d16:1_23:0)	4.64E+10	ND	ND	ND	ND	ND	ND	4.64E+10	ND	ND	ND
Cer(d16:1_24:1)	6.65E+10	ND	ND	ND	3.04E+08	ND	ND	6.65E+10	3.04E+08	ND	ND
Cer(d16:1_24:2)	ND	ND	2.42E+10	ND	ND	ND	ND	ND	ND	2.42E+10	ND
Cer(d16:2_21:1)	ND	ND	1.25E+10	ND	ND	ND	ND	ND	ND	1.25E+10	ND
Cer(d18:1_14:0)	3.15E+10	ND	2.41E+10	ND	2.88E+08	ND	4.69E+08	3.15E+10	2.88E+08	2.41E+10	4.69E+08
Cer(d18:1_16:0)	1.03E+11	ND	1.28E+11	ND	2.23E+08	3.01E+08	ND	1.03E+11	2.23E+08	1.28E+11	0.00E+00
Cer(d18:1_20:0)	ND	ND	2.46E+10	ND	ND	ND	ND	ND	ND	2.46E+10	ND
Cer(d18:1_21:0)	ND	ND	2.87E+09	ND	ND	ND	ND	ND	ND	2.87E+09	ND
Cer(d18:1_22:0)	ND	ND	8.59E+10	ND	1.11E+09	8.98E+08	2.31E+09	ND	1.11E+09	8.68E+10	2.31E+09
Cer(d18:1_22:1)	ND	ND	2.12E+10	ND	ND	ND	ND	ND	ND	2.12E+10	ND
Cer(d18:1_23:0)	ND	1.68E+10	3.22E+10	1.21E+10	7.00E+08	7.35E+08	1.55E+09	ND	1.75E+10	3.29E+10	1.37E+10
Cer(d18:1_24:0)	ND	1.50E+11	1.45E+11	ND	1.54E+09	1.31E+09	3.14E+09	ND	1.51E+11	1.47E+11	3.14E+09
Cer(d18:1_24:1)	2.48E+11	ND	1.44E+11	ND	1.39E+09	1.30E+09	3.15E+09	2.48E+11	1.39E+09	1.45E+11	3.15E+09
Cer(d18:1_24:2)	5.87E+10	ND	7.32E+10	ND	3.89E+08	ND	9.24E+08	5.87E+10	3.89E+08	7.32E+10	9.24E+08

Cer(d18:1_25:1)	ND	ND	1.34E+10	ND	ND	ND	ND	ND	ND	1.34E+10	ND
Cer(d18:1_26:0)	ND	1.19E+10	ND	ND	ND	ND	ND	ND	1.19E+10	ND	ND
Cer(d18:1_26:1)	ND	ND	4.10E+09	ND	ND	ND	ND	ND	ND	4.10E+09	ND
Cer(t16:0_20:0+O)	ND	ND	ND	ND	ND	5.21E+09	ND	ND	ND	5.21E+09	ND
CL(18:2_18:1_18:2_18:2)	ND	3.10E+10	ND	ND	ND	ND	ND	ND	3.10E+10	ND	ND
CL(18:2_18:2_18:2_18:1)	ND	ND	ND	2.97E+10	ND	ND	ND	ND	ND	ND	2.97E+10
CL(18:2_18:2_18:2_18:2)	2.71E+10	ND	ND	ND	ND	ND	ND	2.71E+10	ND	ND	ND
CL(20:5_18:2_18:2_18:2)	6.88E+09	ND	ND	ND	ND	ND	ND	6.88E+09	ND	ND	ND
CmE(2:0)	ND	ND	ND	ND	2.64E+09	ND	ND	ND	2.64E+09	ND	ND
DG(14:0_18:2)	ND	ND	ND	ND	8.23E+10	ND	8.49E+10	ND	8.23E+10	ND	8.49E+10
DG(14:0_18:3)	ND	ND	ND	ND	3.89E+08	ND	ND	ND	3.89E+08	ND	ND
DG(16:0_18:1)	ND	ND	ND	ND	1.10E+10	1.26E+10	2.30E+10	ND	1.10E+10	1.26E+10	2.30E+10
DG(16:0_18:3)	ND	ND	ND	ND	3.74E+09	5.11E+09	5.75E+09	ND	3.74E+09	5.11E+09	5.75E+09
DG(16:0_20:4)	ND	ND	ND	ND	5.00E+09	4.64E+09	9.03E+09	ND	5.00E+09	4.64E+09	9.03E+09
DG(16:0_22:4)	ND	ND	ND	ND	4.76E+09	7.25E+09	8.95E+09	ND	4.76E+09	7.25E+09	8.95E+09
DG(16:0_22:6)	2.48E+11	ND	ND	ND	ND	ND	9.99E+09	2.48E+11	ND	ND	9.99E+09
DG(16:1_18:1)	ND	ND	ND	ND	2.98E+10	3.74E+10	5.22E+10	ND	2.98E+10	3.74E+10	5.22E+10
DG(16:1_18:2)	ND	ND	ND	ND	6.86E+09	9.93E+09	1.17E+10	ND	6.86E+09	9.93E+09	1.17E+10
DG(16:1_18:3)	ND	ND	ND	ND	1.31E+09	2.13E+09	2.45E+09	ND	1.31E+09	2.13E+09	2.45E+09
DG(18:0_16:0)	ND	ND	ND	ND	ND	4.74E+09	4.64E+09	ND	ND	4.74E+09	4.64E+09
DG(18:0_18:1)	ND	ND	ND	ND	ND	ND	6.34E+09	ND	ND	ND	6.34E+09
DG(18:0_18:2)	ND	ND	ND	ND	7.44E+09	9.48E+09	1.48E+10	ND	7.44E+09	9.48E+09	1.48E+10
DG(18:0_20:4)	ND	ND	ND	ND	6.68E+09	8.12E+09	1.40E+10	ND	6.68E+09	8.12E+09	1.40E+10
DG(18:0_22:5)	ND	ND	ND	ND	ND	ND	4.33E+09	ND	ND	ND	4.33E+09
DG(18:0_22:6)	ND	ND	ND	ND	ND	ND	5.60E+09	ND	ND	ND	5.60E+09
DG(18:1_18:1)	ND	ND	ND	ND	2.80E+10	3.63E+10	5.16E+10	ND	2.80E+10	3.63E+10	5.16E+10
DG(18:1_18:2)	2.53E+10	ND	ND	ND	8.66E+10	1.11E+11	1.43E+11	2.53E+10	8.66E+10	1.11E+11	1.43E+11
DG(18:1_18:3)	ND	ND	ND	ND	ND	1.29E+10	ND	ND	ND	1.29E+10	ND
DG(18:1_20:2)	ND	ND	ND	ND	7.94E+09	1.10E+10	1.38E+10	ND	7.94E+09	1.10E+10	1.38E+10
DG(18:1_20:4)	ND	ND	ND	ND	8.77E+09	1.28E+10	1.45E+10	ND	8.77E+09	1.28E+10	1.45E+10
DG(18:1_22:4)	ND	ND	ND	ND	7.83E+09	1.35E+10	1.37E+10	ND	7.83E+09	1.35E+10	1.37E+10
DG(18:1_22:5)	ND	ND	ND	ND	7.43E+09	ND	1.30E+10	ND	7.43E+09	ND	1.30E+10
DG(18:1_22:6)	ND	ND	ND	ND	6.75E+09	1.43E+10	1.54E+10	ND	6.75E+09	1.43E+10	1.54E+10
DG(18:2_18:2)	ND	ND	ND	ND	4.14E+10	5.46E+10	7.98E+10	ND	4.14E+10	5.46E+10	7.98E+10

DG(18:2_20:4)	ND	ND	ND	ND	3.69E+09	4.56E+09	6.47E+09	ND	3.69E+09	4.56E+09	6.47E+09
DG(18:2_22:6)	ND	ND	ND	ND	4.64E+09	ND	1.15E+10	ND	4.64E+09	ND	1.15E+10
DG(18:3_18:2)	ND	ND	ND	ND	7.61E+09	1.00E+10	1.54E+10	ND	7.61E+09	1.00E+10	1.54E+10
DG(18:3_18:3)	ND	ND	ND	ND	ND	6.00E+08	9.19E+08	ND	ND	6.00E+08	9.19E+08
DG(18:4_16:0)	ND	ND	ND	ND	1.00E+09	ND	1.82E+09	ND	1.00E+09	ND	1.82E+09
DG(18:4_18:1)	ND	ND	ND	ND	1.27E+09	1.64E+09	1.96E+09	ND	1.27E+09	1.64E+09	1.96E+09
DG(20:1_18:1)	ND	ND	ND	ND	ND	4.47E+09	5.68E+09	ND	ND	4.47E+09	5.68E+09
DG(20:2_18:2)	ND	ND	ND	ND	3.83E+09	6.06E+09	8.12E+09	ND	3.83E+09	6.06E+09	8.12E+09
DG(20:3_18:2)	ND	ND	ND	ND	5.64E+09	8.71E+09	1.04E+10	ND	5.64E+09	8.71E+09	1.04E+10
DG(22:4_18:2)	ND	ND	ND	ND	4.72E+09	8.01E+09	9.37E+09	ND	4.72E+09	8.01E+09	9.37E+09
DG(22:5_18:2)	ND	ND	ND	ND	ND	ND	1.03E+10	ND	ND	ND	1.03E+10
dMePE(16:0_18:2)	ND	ND	3.58E+11	ND	ND	ND	ND	ND	ND	3.58E+11	ND
dMePE(16:0_22:5)	ND	ND	1.90E+11	ND	ND	ND	ND	ND	ND	1.90E+11	ND
FA(18:4)	ND	4.19E+10	ND	5.84E+10	ND	ND	ND	ND	4.19E+10	ND	5.84E+10
FA(20:4)	ND	8.86E+12	ND	1.22E+13	3.36E+10	ND	ND	ND	8.90E+12	ND	1.22E+13
FA(20:5)	ND	3.04E+11	4.07E+11	2.70E+11	ND	ND	4.05E+09	ND	3.04E+11	4.07E+11	2.74E+11
FA(22:4)	ND	ND	1.42E+12	ND	ND	ND	ND	ND	ND	1.42E+12	ND
FA(22:5)	ND	3.41E+12	5.12E+12	4.84E+12	6.42E+09	ND	ND	ND	3.42E+12	5.12E+12	4.84E+12
FA(22:6)	ND	3.31E+12	4.28E+12	ND	1.19E+10	ND	3.18E+10	ND	3.32E+12	4.28E+12	3.18E+10
LPC(16:0)	ND	4.50E+12	4.77E+12	4.21E+12	6.41E+09	8.69E+09	9.43E+09	ND	4.51E+12	4.78E+12	4.22E+12
LPC(16:0e)	ND	ND	6.04E+10	ND	ND	ND	ND	ND	ND	6.04E+10	ND
LPC(16:1)	ND	ND	2.76E+11	5.39E+11	ND	ND	5.89E+08	ND	ND	2.76E+11	5.40E+11
LPC(17:1)	ND	ND	9.73E+10	ND	ND	ND	ND	ND	ND	9.73E+10	ND
LPC(18:0)	7.36E+09	ND	ND	ND	ND	ND	ND	7.36E+09	ND	ND	ND
LPC(18:1)	ND	ND	4.26E+12	4.38E+12	ND	ND	5.49E+09	ND	ND	4.26E+12	4.38E+12
LPC(18:2)	2.19E+11	8.32E+11	7.45E+12	8.68E+12	6.77E+09	6.45E+09	8.91E+09	2.19E+11	8.39E+11	7.45E+12	8.69E+12
LPC(18:3)	ND	7.01E+10	4.32E+10	ND	1.09E+09	1.35E+09	1.74E+09	ND	7.12E+10	4.45E+10	1.74E+09
LPC(20:1)	ND	9.70E+10	ND	ND	ND	ND	ND	ND	9.70E+10	ND	ND
LPC(20:3)	ND	ND	1.76E+12	1.46E+12	5.09E+09	ND	ND	ND	5.09E+09	1.76E+12	1.46E+12
LPC(20:4)	ND	ND	1.37E+12	2.85E+12	ND	6.88E+08	ND	ND	ND	1.37E+12	2.85E+12
LPC(20:5)	ND	2.70E+10	1.30E+12	1.77E+12	ND	ND	ND	ND	2.70E+10	1.30E+12	1.77E+12
LPC(22:4)	ND	ND	3.63E+11	ND	ND	ND	ND	ND	ND	3.63E+11	ND
LPC(22:5)	ND	ND	2.02E+12	ND	ND	ND	ND	ND	ND	2.02E+12	ND
LPC(22:6)	ND	ND	1.03E+11	4.28E+11	7.58E+08	2.06E+09	1.54E+09	ND	7.58E+08	1.05E+11	4.29E+11

LPE(18:0)	ND	ND	1.45E+11	ND	ND	ND	ND	ND	ND	1.45E+11	ND
LPE(18:2)	ND	ND	3.96E+10	ND	ND	ND	ND	ND	ND	3.96E+10	ND
LPE(18:3e)	ND	1.14E+10	ND	ND	ND	ND	ND	ND	1.14E+10	ND	ND
LPE(20:4)	ND	ND	3.16E+10	ND	ND	ND	ND	ND	ND	3.16E+10	ND
LPE(22:5)	ND	ND	2.01E+11	ND	ND	ND	ND	ND	ND	2.01E+11	ND
LPE(22:6)	ND	ND	1.57E+11	2.30E+11	ND	ND	ND	ND	ND	1.57E+11	2.30E+11
LPG(18:2)	ND	2.50E+09	3.07E+09	1.93E+09	ND	ND	ND	ND	2.50E+09	3.07E+09	1.93E+09
LPI(20:4)	1.22E+10	1.13E+10	1.62E+10	2.10E+10	ND	ND	ND	1.22E+10	1.13E+10	1.62E+10	2.10E+10
LPS(20:4)	1.39E+09	ND	ND	2.58E+09	ND	ND	ND	1.39E+09	ND	ND	2.58E+09
LPS(22:5)	ND	6.05E+09	ND	ND	ND	ND	ND	ND	6.05E+09	ND	ND
MG(18:3)	ND	2.91E+09	ND	ND	ND	ND	ND	ND	2.91E+09	ND	ND
PC(15:0_20:4)	7.46E+11	ND	ND	ND	ND	ND	ND	7.46E+11	ND	ND	ND
PC(16:0_16:0)	ND	ND	ND	ND	1.86E+09	3.90E+09	3.09E+09	ND	1.86E+09	3.90E+09	3.09E+09
PC(16:0_18:1)	ND	ND	ND	ND	7.48E+09	1.14E+10	1.15E+10	ND	7.48E+09	1.14E+10	1.15E+10
PC(16:0_18:2)	1.13E+12	ND	3.75E+11	ND	4.30E+09	6.80E+09	5.24E+09	1.13E+12	4.30E+09	3.81E+11	5.24E+09
PC(16:0_20:3)	ND	ND	ND	ND	8.33E+08	ND	ND	ND	8.33E+08	ND	ND
PC(16:0_20:4)	ND	ND	ND	ND	6.76E+08	ND	1.17E+09	ND	6.76E+08	ND	1.17E+09
PC(16:0_22:5)	ND	ND	3.23E+10	ND	4.09E+08	ND	ND	ND	4.09E+08	3.23E+10	ND
PC(16:0_22:6)	6.30E+11	ND	ND	ND	3.33E+08	ND	ND	6.30E+11	3.33E+08	ND	ND
PC(16:0e_18:1)	ND	2.22E+11	ND	ND	ND	ND	4.64E+08	ND	2.22E+11	ND	4.64E+08
PC(16:0e_22:4)	ND	2.19E+12	ND	ND	ND	ND	ND	ND	2.19E+12	ND	ND
PC(16:0e_22:5)	3.19E+11	ND	ND	ND	3.11E+08	ND	3.50E+08	3.19E+11	3.11E+08	ND	3.50E+08
PC(16:0e_22:6)	3.10E+11	ND	ND	ND	ND	ND	ND	3.10E+11	ND	ND	ND
PC(16:1e_22:5)	4.58E+12	ND	ND	ND	ND	ND	ND	4.58E+12	ND	ND	ND
PC(18:0_18:1)	5.73E+11	ND	ND	ND	1.06E+09	ND	ND	5.73E+11	1.06E+09	ND	ND
PC(18:1_18:1)	ND	ND	ND	ND	1.77E+09	ND	ND	ND	1.77E+09	ND	ND
PC(18:1_18:2)	ND	ND	ND	ND	1.45E+09	ND	1.51E+09	ND	1.45E+09	ND	1.51E+09
PC(18:1_20:2)	ND	ND	2.59E+11	ND	ND	ND	ND	ND	ND	2.59E+11	ND
PC(18:1e_20:4)	ND	ND	ND	ND	3.85E+08	ND	4.21E+08	ND	3.85E+08	ND	4.21E+08
PC(18:2_18:2)	ND	ND	ND	ND	5.36E+08	ND	ND	ND	5.36E+08	ND	ND
PC(18:3_18:2)	ND	ND	ND	1.33E+11	ND	ND	ND	ND	ND	ND	1.33E+11
PE(16:0_18:1)	3.93E+10	1.64E+10	4.31E+10	ND	ND	ND	ND	3.93E+10	1.64E+10	4.31E+10	ND
PE(16:0_18:2)	4.52E+10	ND	2.70E+10	ND	ND	ND	ND	4.52E+10	ND	2.70E+10	ND
PE(16:0_20:4)	9.18E+10	ND	ND	ND	ND	ND	ND	9.18E+10	ND	ND	ND

PE(16:0_22:5)	ND	ND	4.18E+10	ND	ND	ND	ND	ND	ND	4.18E+10	ND
PE(16:1e_18:1)	1.59E+11	ND	ND	ND	ND	ND	ND	1.59E+11	ND	ND	ND
PE(16:1e_18:2)	5.61E+10	ND	ND	ND	ND	ND	ND	5.61E+10	ND	ND	ND
PE(16:1e_20:4)	ND	3.21E+11	ND	2.19E+11	ND	ND	ND	ND	3.21E+11	ND	2.19E+11
PE(18:0_18:1)	2.91E+10	ND	ND	ND	ND	ND	ND	2.91E+10	ND	ND	ND
PE(18:0_20:4)	1.01E+12	ND	3.52E+11	ND	6.17E+08	2.46E+09	3.74E+09	1.01E+12	6.17E+08	3.54E+11	3.74E+09
PE(18:0_22:4)	ND	ND	2.40E+12	ND	ND	ND	3.74E+08	ND	ND	2.40E+12	3.74E+08
PE(18:0_22:5)	ND	ND	2.22E+11	ND	4.45E+08	1.99E+09	2.70E+09	ND	4.45E+08	2.24E+11	2.70E+09
PE(18:0p_17:1)	ND	ND	1.08E+11	ND	ND	ND	ND	ND	ND	1.08E+11	ND
PE(18:1_22:5)	ND	ND	9.10E+10	ND	ND	ND	ND	ND	ND	9.10E+10	ND
PE(18:1e_20:4)	3.97E+11	ND	6.04E+11	ND	ND	ND	ND	3.97E+11	ND	6.04E+11	ND
PE(18:1p_18:1)	4.65E+11	ND	ND	ND	ND	ND	ND	4.65E+11	ND	ND	ND
PE(18:1p_22:5)	ND	ND	2.08E+12	ND	ND	ND	ND	ND	ND	2.08E+12	ND
PE(18:2_22:6)	1.31E+10	ND	1.15E+10	ND	ND	ND	ND	1.31E+10	ND	1.15E+10	ND
PE(18:2e_18:2)	6.16E+10	ND	1.29E+11	ND	ND	ND	ND	6.16E+10	ND	1.29E+11	ND
PE(18:2e_20:4)	3.83E+11	ND	ND	ND	ND	ND	ND	3.83E+11	ND	ND	ND
PE(18:2e_22:6)	9.30E+11	ND	ND	ND	ND	ND	ND	9.30E+11	ND	ND	ND
PE(18:3e_20:4)	ND	ND	ND	9.47E+11	ND	ND	ND	ND	ND	ND	9.47E+11
PE(18:3e_22:6)	3.50E+10	1.91E+10	7.24E+10	ND	ND	ND	ND	3.50E+10	1.91E+10	7.24E+10	ND
PEt(15:0_18:1)	ND	ND	ND	ND	3.50E+09	7.38E+09	7.06E+09	ND	3.50E+09	7.38E+09	7.06E+09
PG(28:0_16:0)	ND	ND	ND	ND	ND	ND	1.94E+11	ND	ND	ND	1.94E+11
PG(28:0_18:1)	1.39E+12	ND	ND	ND	2.96E+11	ND	3.89E+11	1.39E+12	2.96E+11	ND	ND
PG(28:0_18:2)	ND	ND	ND	ND	1.00E+11	1.14E+11	ND	ND	1.00E+11	1.14E+11	ND
PG(28:0_18:3)	ND	ND	ND	ND	ND	ND	2.01E+10	ND	ND	ND	2.01E+10
PG(28:1_16:0)	ND	ND	ND	ND	ND	5.36E+10	ND	ND	ND	5.36E+10	ND
PG(30:0_18:2)	ND	ND	ND	ND	ND	ND	1.87E+11	ND	ND	ND	1.87E+11
PI(18:0_20:4)	ND	4.22E+11	1.35E+11	ND	9.76E+08	1.58E+09	1.01E+09	ND	4.23E+11	1.36E+11	1.01E+09
PI(18:0_22:6)	ND	3.45E+10	3.28E+10	ND	ND	ND	ND	ND	3.45E+10	3.28E+10	ND
PI(18:1_20:4)	ND	9.42E+10	ND	ND	ND	ND	ND	ND	9.42E+10	ND	ND
PI(18:1_22:6)	2.50E+10	4.86E+09	ND	ND	ND	ND	ND	2.50E+10	4.86E+09	ND	ND
PS(18:0_18:1)	ND	ND	ND	ND	2.02E+08	ND	1.21E+09	ND	2.02E+08	ND	1.21E+09
PS(18:0_18:2)	ND	ND	ND	ND	1.31E+08	ND	ND	ND	1.31E+08	ND	ND
PS(18:0_22:5)	ND	ND	ND	ND	ND	5.82E+08	6.45E+08	ND	ND	5.82E+08	6.45E+08
PS(20:0_22:5)	ND	ND	6.26E+11	ND	ND	ND	ND	ND	ND	6.26E+11	ND

SM(d18:1_22:2)	ND	ND	ND	ND	7.30E+08	ND	ND	ND	7.30E+08	ND	ND
SM(d18:1_24:1)	ND	4.00E+12	ND	ND	ND	ND	ND	ND	4.00E+12	ND	ND
SPH(t16:0)	ND	ND	ND	ND	ND	3.21E+10	2.39E+10	ND	ND	3.21E+10	2.39E+10
SPH(t18:0)	ND	ND	ND	ND	1.41E+10	2.36E+10	ND	ND	1.41E+10	2.36E+10	ND
SPH(t20:0)	ND	ND	ND	ND	1.19E+10	2.25E+10	1.89E+10	ND	1.19E+10	2.25E+10	1.89E+10
TG(12:0_14:0_18:2)	ND	ND	ND	ND	2.50E+10	2.04E+10	4.13E+10	ND	2.50E+10	2.04E+10	4.13E+10
TG(12:0e_6:0_18:2)	ND	ND	ND	ND	4.03E+10	ND	ND	ND	4.03E+10	ND	ND
TG(12:0e_8:0_12:2)	ND	ND	ND	ND	ND	ND	3.26E+10	ND	ND	ND	3.26E+10
TG(14:0_18:2_18:3)	ND	9.94E+12	ND	ND	ND	ND	ND	ND	9.94E+12	ND	ND
TG(14:0_18:2_20:5)	3.37E+12	ND	ND	ND	7.24E+10	1.01E+11	1.31E+11	3.37E+12	7.24E+10	1.01E+11	1.31E+11
TG(14:0_18:3_20:5)	ND	ND	1.03E+12	ND	ND	ND	ND	ND	ND	1.03E+12	ND
TG(14:1e_10:1_10:1)	ND	ND	ND	ND	ND	5.52E+09	ND	ND	ND	5.52E+09	ND
TG(15:0_14:0_16:0)	ND	ND	ND	ND	ND	1.67E+10	2.21E+10	ND	ND	1.67E+10	2.21E+10
TG(15:0_14:0_18:2)	ND	ND	ND	ND	7.71E+10	1.40E+11	ND	ND	7.71E+10	1.40E+11	ND
TG(15:0_16:0_16:0)	ND	ND	ND	ND	6.72E+10	1.02E+11	1.45E+11	ND	6.72E+10	1.02E+11	1.45E+11
TG(15:0_16:0_16:1)	ND	ND	ND	ND	8.55E+10	1.33E+11	1.85E+11	ND	8.55E+10	1.33E+11	1.85E+11
TG(15:0_16:0_18:1)	ND	ND	ND	ND	4.98E+11	6.59E+11	8.48E+11	ND	4.98E+11	6.59E+11	8.48E+11
TG(15:0_16:0_20:4)	ND	ND	ND	ND	1.80E+10	2.73E+10	2.74E+10	ND	1.80E+10	2.73E+10	2.74E+10
TG(15:0_16:1_18:1)	ND	ND	ND	ND	5.50E+11	7.28E+11	8.53E+11	ND	5.50E+11	7.28E+11	8.53E+11
TG(15:0_16:1_18:2)	ND	ND	ND	ND	1.17E+11	4.29E+11	2.52E+11	ND	1.17E+11	4.29E+11	2.52E+11
TG(15:0_18:1_18:1)	ND	ND	ND	ND	1.37E+12	2.05E+12	1.67E+12	ND	1.37E+12	2.05E+12	1.67E+12
TG(15:0_18:1_18:2)	ND	ND	ND	ND	9.62E+11	1.37E+12	1.31E+12	ND	9.62E+11	1.37E+12	1.31E+12
TG(15:0_18:1_20:4)	ND	ND	ND	ND	5.39E+10	ND	9.25E+10	ND	5.39E+10	ND	9.25E+10
TG(15:0_18:1_20:5)	ND	ND	ND	ND	3.64E+10	6.24E+10	ND	ND	3.64E+10	6.24E+10	ND
TG(15:0_18:2_18:2)	ND	ND	ND	ND	4.32E+11	8.35E+11	7.41E+11	ND	4.32E+11	8.35E+11	7.41E+11
TG(15:0_18:2_18:3)	ND	ND	ND	ND	8.71E+10	1.82E+11	1.55E+11	ND	8.71E+10	1.82E+11	1.55E+11
TG(15:0_18:2_20:5)	ND	ND	ND	ND	2.22E+10	3.91E+10	3.28E+10	ND	2.22E+10	3.91E+10	3.28E+10
TG(16:0_11:3_16:0)	ND	ND	ND	ND	1.01E+10	ND	ND	ND	1.01E+10	ND	ND
TG(16:0_12:0_14:0)	ND	ND	ND	ND	1.27E+10	ND	2.54E+10	ND	1.27E+10	ND	2.54E+10
TG(16:0_14:0_14:0)	ND	3.38E+11	ND	ND	1.10E+11	6.81E+10	2.08E+11	ND	4.48E+11	6.81E+10	2.08E+11
TG(16:0_14:0_16:0)	ND	ND	ND	ND	3.93E+11	2.88E+11	6.32E+11	ND	3.93E+11	2.88E+11	6.32E+11
TG(16:0_14:0_16:1)	ND	2.54E+12	ND	ND	6.26E+11	5.07E+11	1.19E+12	ND	3.16E+12	5.07E+11	1.19E+12
TG(16:0_14:0_18:1)	ND	ND	2.46E+12	4.61E+11	2.59E+12	2.34E+12	4.10E+12	ND	2.59E+12	4.80E+12	4.56E+12
TG(16:0_14:0_18:2)	ND	ND	ND	ND	2.26E+12	2.11E+12	3.76E+12	ND	2.26E+12	2.11E+12	3.76E+12

TG(16:0_14:0_20:4)	ND	ND	ND	ND	6.82E+10	7.41E+10	9.87E+10	ND	6.82E+10	7.41E+10	9.87E+10
TG(16:0_16:0_16:0)	ND	ND	ND	1.48E+12	6.10E+11	5.55E+11	9.59E+11	ND	6.10E+11	5.55E+11	2.44E+12
TG(16:0_16:0_17:0)	ND	ND	ND	1.45E+11	1.57E+11	2.68E+11	3.55E+11	ND	1.57E+11	2.68E+11	5.00E+11
TG(16:0_16:0_18:1)	8.94E+13	ND	2.93E+13	ND	5.09E+12	5.14E+12	7.23E+12	8.94E+13	5.09E+12	3.44E+13	7.23E+12
TG(16:0_16:0_18:3)	ND	ND	ND	ND	5.66E+11	ND	ND	ND	5.66E+11	ND	ND
TG(16:0_16:0_20:4)	ND	ND	ND	ND	ND	5.92E+11	8.76E+11	ND	ND	5.92E+11	8.76E+11
TG(16:0_16:0_21:0)	ND	9.61E+10	ND	ND	9.38E+10	1.16E+11	1.87E+11	ND	1.90E+11	1.16E+11	1.87E+11
TG(16:0_16:0_22:5)	ND	ND	ND	ND	ND	1.15E+11	1.66E+11	ND	ND	1.15E+11	1.66E+11
TG(16:0_16:0_22:6)	ND	ND	ND	ND	ND	ND	2.01E+11	ND	ND	ND	2.01E+11
TG(16:0_16:0_23:0)	ND	ND	ND	ND	5.82E+10	6.03E+10	9.47E+10	ND	5.82E+10	6.03E+10	9.47E+10
TG(16:0_16:1_18:1)	3.24E+12	3.98E+11	8.88E+12	ND	6.67E+12	7.11E+12	9.63E+12	3.24E+12	7.07E+12	1.60E+13	9.63E+12
TG(16:0_16:1_18:2)	ND	1.20E+13	ND	ND	ND	ND	ND	ND	1.20E+13	ND	ND
TG(16:0_16:1_18:3)	3.96E+11	1.13E+13	ND	ND	3.05E+11	ND	ND	3.96E+11	1.16E+13	ND	ND
TG(16:0_17:0_18:1)	ND	ND	7.87E+11	ND	9.71E+11	1.07E+12	1.72E+12	ND	9.71E+11	1.07E+12	1.72E+12
TG(16:0_17:0_20:4)	ND	ND	ND	ND	5.73E+10	8.16E+10	9.45E+10	ND	5.73E+10	8.16E+10	9.45E+10
TG(16:0_17:1_18:1)	ND	ND	ND	ND	ND	ND	ND	ND	ND	7.87E+11	ND
TG(16:0_18:1_18:1)	ND	ND	ND	ND	8.21E+12	1.18E+13	1.27E+13	ND	8.21E+12	1.18E+13	1.27E+13
TG(16:0_18:1_18:3)	ND	ND	ND	ND	1.06E+11	6.89E+10	ND	ND	1.06E+11	6.89E+10	ND
TG(16:0_18:1_19:0)	ND	1.57E+12	ND	ND	5.55E+11	7.73E+11	9.49E+11	ND	2.12E+12	7.73E+11	9.49E+11
TG(16:0_18:1_20:1)	ND	ND	ND	ND	5.07E+12	5.12E+12	5.53E+12	ND	5.07E+12	5.12E+12	5.53E+12
TG(16:0_18:1_20:4)	ND	ND	ND	ND	1.51E+12	1.54E+12	1.94E+12	ND	1.51E+12	1.54E+12	1.94E+12
TG(16:0_18:1_20:5)	ND	ND	ND	ND	3.14E+11	3.86E+11	4.09E+11	ND	3.14E+11	3.86E+11	4.09E+11
TG(16:0_18:1_21:0)	ND	1.92E+12	ND	ND	2.79E+11	3.04E+11	3.95E+11	ND	2.20E+12	3.04E+11	3.95E+11
TG(16:0_18:1_22:0)	ND	6.18E+12	ND	ND	6.81E+11	6.72E+11	8.85E+11	ND	6.86E+12	6.72E+11	8.85E+11
TG(16:0_18:1_22:6)	ND	ND	ND	ND	6.99E+11	9.56E+11	1.08E+12	ND	6.99E+11	9.56E+11	1.08E+12
TG(16:0_18:1_23:0)	ND	5.47E+11	ND	ND	1.61E+11	1.42E+11	2.01E+11	ND	7.09E+11	1.42E+11	2.01E+11
TG(16:0_18:1_24:0)	ND	1.54E+12	ND	ND	2.39E+11	2.05E+11	2.98E+11	ND	1.78E+12	2.05E+11	2.98E+11
TG(16:0_18:1_24:1)	1.37E+12	3.85E+12	ND	ND	ND	ND	ND	1.37E+12	3.85E+12	ND	ND
TG(16:0_18:2_18:3)	1.79E+12	ND	ND	ND	1.77E+11	ND	ND	1.79E+12	1.77E+11	ND	ND
TG(16:0_18:2_20:5)	ND	ND	ND	ND	3.13E+11	4.12E+11	4.38E+11	ND	3.13E+11	4.12E+11	4.38E+11
TG(16:0_18:2_23:0)	ND	1.79E+12	ND	ND	2.15E+11	2.73E+11	3.00E+11	ND	2.00E+12	2.73E+11	3.00E+11
TG(16:0_18:3_18:3)	ND	ND	ND	ND	3.31E+10	ND	ND	ND	3.31E+10	ND	ND
TG(16:0_18:3_23:0)	ND	ND	ND	ND	7.30E+09	ND	ND	ND	7.30E+09	ND	ND
TG(16:0_20:1_24:0)	ND	ND	ND	ND	3.40E+10	ND	4.09E+10	ND	3.40E+10	ND	4.09E+10

TG(16:0_20:3_22:0)	ND	ND	ND	ND	ND	9.76E+09	ND	ND	ND	9.76E+09	ND
TG(16:0_20:3_23:0)	ND	ND	ND	ND	2.35E+09	ND	ND	ND	2.35E+09	ND	ND
TG(16:0_20:4_21:0)	ND	2.66E+11	ND	ND	3.22E+10	ND	4.22E+10	ND	2.98E+11	ND	4.22E+10
TG(16:0_20:4_23:0)	ND	ND	ND	ND	1.95E+10	ND	2.29E+10	ND	1.95E+10	ND	2.29E+10
TG(16:0_20:4_24:0)	ND	ND	ND	ND	2.87E+10	ND	ND	ND	2.87E+10	ND	ND
TG(16:0_20:5_21:0)	ND	ND	ND	ND	2.85E+10	5.87E+10	5.65E+10	ND	2.85E+10	5.87E+10	5.65E+10
TG(16:0_20:5_23:0)	ND	ND	ND	ND	ND	2.32E+10	2.80E+10	ND	ND	2.32E+10	2.80E+10
TG(16:0_20:5_24:0)	ND	ND	ND	ND	3.29E+10	ND	2.76E+10	ND	3.29E+10	ND	2.76E+10
TG(16:0_22:0_24:0)	ND	ND	ND	ND	9.81E+08	ND	ND	ND	9.81E+08	ND	ND
TG(16:0_6:0_12:1)	ND	ND	ND	ND	1.65E+10	9.57E+09	2.06E+10	ND	1.65E+10	9.57E+09	2.06E+10
TG(16:0_8:0_10:1)	ND	ND	ND	ND	ND	ND	4.24E+09	ND	ND	ND	4.24E+09
TG(16:0_9:0_9:0)	ND	ND	ND	ND	1.25E+10	8.67E+09	1.32E+10	ND	1.25E+10	8.67E+09	1.32E+10
TG(16:0e_14:0_16:0)	ND	ND	ND	ND	ND	ND	6.87E+09	ND	ND	ND	6.87E+09
TG(16:0e_16:0_16:0)	ND	ND	ND	ND	9.07E+09	6.18E+09	1.69E+10	ND	9.07E+09	6.18E+09	1.69E+10
TG(16:0e_16:0_18:0)	ND	ND	ND	ND	4.56E+09	3.68E+09	8.51E+09	ND	4.56E+09	3.68E+09	8.51E+09
TG(16:0e_16:0_18:1)	ND	ND	ND	ND	4.16E+10	2.83E+10	6.11E+10	ND	4.16E+10	2.83E+10	6.11E+10
TG(16:0e_16:0_22:1)	ND	ND	ND	ND	2.88E+09	ND	ND	ND	2.88E+09	ND	ND
TG(16:0e_16:0_22:4)	ND	ND	ND	ND	7.27E+09	ND	9.22E+09	ND	7.27E+09	ND	9.22E+09
TG(16:0e_18:1_18:2)	ND	ND	ND	ND	2.12E+10	1.68E+10	2.48E+10	ND	2.12E+10	1.68E+10	2.48E+10
TG(16:0e_18:1_22:4)	ND	ND	ND	ND	2.31E+10	1.57E+10	2.36E+10	ND	2.31E+10	1.57E+10	2.36E+10
TG(16:0e_18:1_22:5)	ND	ND	ND	ND	2.10E+10	1.86E+10	2.32E+10	ND	2.10E+10	1.86E+10	2.32E+10
TG(16:0e_18:2_22:4)	ND	ND	ND	ND	1.87E+10	ND	ND	ND	1.87E+10	ND	ND
TG(16:1_12:0_18:2)	ND	ND	ND	ND	4.31E+10	ND	ND	ND	4.31E+10	ND	ND
TG(16:1_14:0_14:0)	ND	4.20E+11	ND	ND	7.08E+10	5.34E+10	1.27E+11	ND	4.91E+11	5.34E+10	1.27E+11
TG(16:1_14:0_16:1)	ND	1.28E+12	ND	ND	2.33E+11	2.30E+11	4.24E+11	ND	1.51E+12	2.30E+11	4.24E+11
TG(16:1_14:0_18:2)	1.79E+11	ND	ND	ND	ND	ND	ND	1.79E+11	ND	ND	ND
TG(16:1_14:0_20:5)	ND	ND	ND	ND	ND	ND	3.92E+10	ND	ND	ND	3.92E+10
TG(16:1_14:1_18:2)	ND	ND	ND	ND	ND	ND	1.43E+11	ND	ND	ND	1.43E+11
TG(16:1_16:1_16:1)	ND	ND	ND	ND	4.50E+11	6.00E+11	1.22E+12	ND	4.50E+11	6.00E+11	1.22E+12
TG(16:1_16:1_18:1)	ND	ND	ND	ND	4.19E+12	4.25E+12	6.02E+12	ND	4.19E+12	4.25E+12	6.02E+12
TG(16:1_16:1_18:2)	ND	ND	ND	ND	1.25E+12	2.08E+12	2.64E+12	ND	1.25E+12	2.08E+12	2.64E+12
TG(16:1_18:1_18:2)	7.93E+12	8.45E+13	ND	ND	6.27E+12	1.10E+13	1.03E+13	7.93E+12	9.08E+13	1.10E+13	1.03E+13
TG(16:1_18:2_18:2)	ND	ND	ND	ND	ND	4.51E+11	ND	ND	ND	4.51E+11	ND
TG(16:1_18:2_18:3)	ND	1.46E+13	ND	ND	ND	ND	ND	ND	1.54E+13	ND	1.31E+12

TG(16:1_18:2_20:5)	ND	ND	ND	ND	8.15E+11	ND	1.31E+12	ND	5.19E+10	8.94E+10	ND
TG(16:1_18:3_18:3)	ND	ND	9.59E+11	4.68E+12	5.19E+10	8.94E+10	ND	ND	ND	9.59E+11	4.68E+12
TG(17:0_18:1_18:1)	ND	ND	6.76E+11	ND	1.10E+12	1.73E+12	1.77E+12	ND	1.10E+12	2.40E+12	1.77E+12
TG(17:0_18:1_18:2)	ND	ND	ND	ND	1.04E+12	1.84E+12	1.62E+12	ND	1.04E+12	1.84E+12	1.62E+12
TG(17:0_18:1_20:3)	ND	ND	ND	ND	5.04E+10	7.01E+10	7.91E+10	ND	5.04E+10	7.01E+10	7.91E+10
TG(17:0_18:1_20:4)	ND	ND	ND	ND	7.72E+10	1.13E+11	1.14E+11	ND	7.72E+10	1.13E+11	1.14E+11
TG(17:0_18:1_22:4)	ND	ND	ND	ND	ND	1.04E+11	ND	ND	ND	1.04E+11	ND
TG(18:0_16:0_16:0)	ND	ND	1.25E+12	ND	1.09E+12	9.79E+11	1.60E+12	ND	1.09E+12	2.23E+12	1.60E+12
TG(18:0_16:0_17:0)	ND	ND	ND	ND	1.52E+11	2.33E+11	2.59E+11	ND	1.52E+11	2.33E+11	2.59E+11
TG(18:0_16:0_18:0)	1.16E+13	ND	1.30E+12	1.70E+12	7.00E+11	7.71E+11	1.18E+12	1.16E+13	7.00E+11	2.08E+12	2.88E+12
TG(18:0_16:0_18:1)	ND	ND	ND	ND	5.75E+12	5.49E+12	8.02E+12	ND	5.75E+12	5.49E+12	8.02E+12
TG(18:0_16:0_18:3)	ND	ND	ND	ND	1.10E+13	1.20E+13	1.48E+13	ND	1.10E+13	1.20E+13	1.48E+13
TG(18:0_16:0_20:0)	1.92E+12	1.24E+12	9.52E+11	2.16E+12	2.71E+11	2.60E+11	3.91E+11	1.92E+12	1.52E+12	1.21E+12	2.55E+12
TG(18:0_16:0_20:1)	ND	ND	ND	ND	1.82E+12	1.98E+12	2.54E+12	ND	1.82E+12	1.98E+12	2.54E+12
TG(18:0_16:0_20:3)	ND	ND	ND	ND	4.42E+10	4.39E+10	6.17E+10	ND	4.42E+10	4.39E+10	6.17E+10
TG(18:0_16:0_20:4)	ND	ND	ND	ND	1.93E+11	2.10E+11	4.48E+11	ND	1.93E+11	2.10E+11	4.48E+11
TG(18:0_16:0_22:0)	ND	ND	ND	3.93E+11	9.89E+10	7.85E+10	1.35E+11	ND	9.89E+10	7.85E+10	5.28E+11
TG(18:0_16:0_22:5)	ND	ND	ND	ND	1.04E+11	1.44E+11	1.73E+11	ND	1.04E+11	1.44E+11	1.73E+11
TG(18:0_16:0_23:0)	ND	ND	ND	1.25E+11	ND	ND	ND	ND	ND	ND	1.25E+11
TG(18:0_16:0_24:0)	ND	ND	ND	ND	2.18E+10	1.90E+10	2.91E+10	ND	2.18E+10	1.90E+10	2.91E+10
TG(18:0_18:0_18:1)	ND	4.36E+12	ND	ND	ND	ND	ND	ND	4.36E+12	ND	ND
TG(18:0_18:0_20:3)	ND	ND	ND	ND	2.41E+10	ND	ND	ND	2.41E+10	ND	ND
TG(18:0_18:0_22:5)	ND	ND	ND	ND	2.18E+10	3.60E+10	4.11E+10	ND	2.18E+10	3.60E+10	4.11E+10
TG(18:0_18:1_18:2)	ND	6.89E+11	ND	ND	ND	ND	ND	ND	6.89E+11	ND	ND
TG(18:0_18:1_20:1)	1.69E+13	1.54E+13	ND	ND	8.16E+11	1.17E+12	1.26E+12	1.69E+13	1.62E+13	1.17E+12	1.26E+12
TG(18:0_18:1_20:3)	ND	ND	ND	ND	ND	1.41E+11	1.69E+11	ND	ND	1.41E+11	1.69E+11
TG(18:0_18:1_20:4)	ND	3.52E+12	ND	ND	2.11E+11	2.95E+11	2.76E+11	ND	3.73E+12	2.95E+11	2.76E+11
TG(18:0_18:1_22:4)	ND	ND	3.04E+12	ND	8.48E+11	1.26E+12	1.24E+12	ND	8.48E+11	4.30E+12	1.24E+12
TG(18:0_18:1_22:5)	ND	2.84E+12	ND	ND	1.64E+11	2.40E+11	2.43E+11	ND	3.00E+12	2.40E+11	2.43E+11
TG(18:0_20:4_22:4)	ND	ND	ND	ND	ND	1.05E+11	ND	ND	ND	1.05E+11	ND
TG(18:0_9:0_9:0)	ND	ND	ND	ND	ND	ND	2.29E+09	ND	ND	ND	2.29E+09
TG(18:1_14:0_20:5)	ND	ND	ND	ND	ND	ND	1.98E+11	ND	ND	ND	1.98E+11
TG(18:1_17:1_18:2)	ND	ND	ND	ND	6.45E+11	1.27E+12	1.06E+12	ND	6.45E+11	1.27E+12	1.06E+12
TG(18:1_17:1_20:4)	ND	ND	ND	ND	6.24E+10	1.01E+11	9.18E+10	ND	6.24E+10	1.01E+11	9.18E+10

TG(18:1_17:1_22:5)	ND	ND	ND	ND	ND	1.44E+11	1.27E+11	ND	ND	1.44E+11	1.27E+11
TG(18:1_18:1_18:1)	ND	1.96E+12	ND	ND	7.74E+12	9.18E+12	1.05E+13	ND	9.70E+12	9.18E+12	1.05E+13
TG(18:1_18:1_18:2)	ND	9.92E+13	ND	ND	9.88E+12	1.13E+13	1.29E+13	ND	1.09E+14	1.13E+13	1.29E+13
TG(18:1_18:1_20:3)	ND	ND	ND	ND	2.98E+12	2.04E+12	3.67E+12	ND	2.98E+12	2.04E+12	3.67E+12
TG(18:1_18:1_20:4)	ND	ND	ND	ND	3.03E+11	3.90E+11	3.88E+11	ND	3.03E+11	3.90E+11	3.88E+11
TG(18:1_18:1_22:4)	ND	ND	ND	ND	1.23E+12	1.76E+12	1.80E+12	ND	1.23E+12	1.76E+12	1.80E+12
TG(18:1_18:1_22:5)	ND	ND	ND	ND	4.93E+11	7.52E+11	8.71E+11	ND	4.93E+11	7.52E+11	8.71E+11
TG(18:1_18:1_22:6)	ND	ND	ND	ND	6.99E+11	1.15E+12	1.26E+12	ND	6.99E+11	1.15E+12	1.26E+12
TG(18:1_18:1_23:0)	ND	5.34E+11	ND	ND	ND	6.81E+10	ND	ND	5.34E+11	6.81E+10	ND
TG(18:1_18:1_24:0)	ND	ND	ND	ND	8.22E+10	ND	ND	ND	8.22E+10	ND	ND
TG(18:1_18:2_18:2)	ND	8.76E+11	ND	ND	8.40E+12	9.92E+12	1.18E+13	ND	9.28E+12	9.92E+12	1.18E+13
TG(18:1_18:2_20:2)	ND	ND	2.95E+12	ND	ND	ND	ND	ND	ND	2.95E+12	ND
TG(18:1_18:2_20:3)	ND	ND	2.70E+12	ND	ND	ND	ND	ND	ND	2.70E+12	ND
TG(18:1_18:2_20:4)	ND	1.55E+12	ND	ND	3.59E+11	4.45E+11	ND	ND	1.91E+12	4.45E+11	ND
TG(18:1_18:2_20:5)	ND	1.24E+13	ND	ND	ND	ND	ND	ND	1.24E+13	ND	ND
TG(18:1_18:2_21:0)	ND	ND	ND	ND	1.61E+11	2.70E+11	ND	ND	1.61E+11	2.70E+11	ND
TG(18:1_18:2_22:0)	ND	2.55E+12	ND	ND	ND	ND	ND	ND	2.55E+12	ND	ND
TG(18:1_18:2_22:1)	ND	4.63E+12	ND	ND	ND	ND	ND	ND	4.63E+12	ND	ND
TG(18:1_18:2_22:4)	ND	1.09E+13	ND	ND	1.14E+12	1.54E+12	1.78E+12	ND	1.21E+13	1.54E+12	1.78E+12
TG(18:1_18:2_22:6)	ND	ND	ND	ND	4.51E+11	6.78E+11	7.21E+11	ND	4.51E+11	6.78E+11	7.21E+11
TG(18:1_18:2_23:0)	ND	ND	ND	ND	ND	8.91E+10	8.56E+10	ND	ND	8.91E+10	8.56E+10
TG(18:1_18:2_24:0)	ND	1.07E+12	ND	ND	ND	ND	1.05E+11	ND	1.07E+12	ND	1.05E+11
TG(18:1_18:2_24:1)	ND	ND	ND	ND	1.08E+11	1.44E+11	1.33E+11	ND	1.08E+11	1.44E+11	1.33E+11
TG(18:1_18:3_22:4)	ND	ND	ND	ND	1.92E+11	ND	ND	ND	1.92E+11	ND	ND
TG(18:1_20:3_22:4)	ND	ND	ND	ND	7.58E+10	1.53E+11	1.70E+11	ND	7.58E+10	1.53E+11	1.70E+11
TG(18:1_20:3_22:6)	ND	ND	ND	ND	2.70E+10	4.89E+10	4.50E+10	ND	2.70E+10	4.89E+10	4.50E+10
TG(18:1_20:4_20:4)	ND	ND	ND	ND	ND	2.49E+11	ND	ND	ND	2.49E+11	ND
TG(18:1_20:4_22:0)	1.20E+12	ND	ND	ND	ND	ND	ND	1.20E+12	ND	ND	ND
TG(18:1_20:4_22:4)	ND	ND	ND	ND	1.24E+11	2.04E+11	1.52E+11	ND	1.24E+11	2.04E+11	1.52E+11
TG(18:1_20:4_23:0)	ND	ND	ND	ND	3.77E+09	ND	ND	ND	3.77E+09	ND	ND
TG(18:1_20:4_24:0)	8.89E+10	ND	ND	ND	ND	ND	ND	8.89E+10	ND	ND	ND
TG(18:1_20:5_24:1)	ND	ND	3.87E+11	ND	ND	ND	ND	ND	ND	3.87E+11	ND
TG(18:1_22:0_22:6)	ND	ND	ND	ND	ND	3.16E+10	4.03E+10	ND	ND	3.16E+10	4.03E+10
TG(18:1_22:3_22:4)	1.06E+11	ND	ND	ND	ND	ND	ND	1.06E+11	ND	ND	ND

TG(18:1_22:6_24:1)	ND	ND	ND	ND	ND	9.56E+09	ND	ND	ND	9.56E+09	ND
TG(18:1e_16:0_18:1)	ND	ND	ND	ND	2.74E+10	2.17E+10	3.27E+10	ND	2.74E+10	2.17E+10	3.27E+10
TG(18:2_13:0_18:2)	ND	ND	ND	ND	ND	1.19E+11	1.12E+11	ND	ND	1.19E+11	1.12E+11
TG(18:2_14:1_18:2)	ND	ND	ND	ND	4.27E+11	ND	7.34E+11	ND	4.27E+11	ND	7.34E+11
TG(18:2_17:1_18:2)	ND	ND	ND	ND	2.38E+11	5.23E+11	4.32E+11	ND	2.38E+11	5.23E+11	4.32E+11
TG(18:2_17:1_20:4)	ND	ND	ND	ND	3.56E+10	5.74E+10	4.48E+10	ND	3.56E+10	5.74E+10	4.48E+10
TG(18:2_18:2_18:2)	ND	ND	ND	ND	4.66E+12	6.02E+12	ND	ND	4.66E+12	6.02E+12	ND
TG(18:2_18:2_22:6)	ND	ND	ND	ND	1.70E+11	3.19E+11	3.54E+11	ND	1.70E+11	3.19E+11	3.54E+11
TG(18:2_18:2_23:1)	ND	ND	4.85E+10	ND	ND	ND	ND	ND	ND	4.85E+10	ND
TG(18:3_14:1_18:2)	ND	ND	ND	5.43E+11	ND	ND	ND	ND	ND	ND	5.43E+11
TG(18:3_18:2_18:2)	ND	2.37E+13	ND	ND	6.67E+11	1.29E+12	1.87E+12	ND	2.44E+13	1.29E+12	1.87E+12
TG(18:3_18:2_18:3)	ND	ND	ND	ND	ND	3.24E+11	3.22E+11	ND	ND	3.24E+11	3.22E+11
TG(18:3_18:2_20:3)	ND	ND	ND	ND	ND	6.72E+11	ND	ND	ND	6.72E+11	ND
TG(18:3_18:2_20:5)	1.00E+13	ND	ND	ND	5.26E+10	1.20E+11	1.49E+11	1.00E+13	5.26E+10	1.20E+11	1.49E+11
TG(18:3_18:2_22:6)	ND	ND	2.77E+12	ND	ND	ND	5.03E+10	ND	ND	2.77E+12	5.03E+10
TG(18:3_18:2_24:2)	ND	ND	ND	ND	1.10E+11	ND	ND	ND	1.10E+11	ND	ND
TG(18:3_18:3_18:3)	ND	ND	ND	ND	3.03E+10	4.99E+10	6.74E+10	ND	3.03E+10	4.99E+10	6.74E+10
TG(18:3_18:3_22:6)	ND	8.17E+11	ND	ND	ND	ND	ND	ND	8.17E+11	ND	ND
TG(18:3_20:5_22:6)	6.91E+11	ND	ND	ND	ND	ND	ND	6.91E+11	ND	ND	ND
TG(18:4_14:0_16:0)	ND	ND	ND	ND	2.45E+10	2.16E+10	4.25E+10	ND	2.45E+10	2.16E+10	4.25E+10
TG(18:4_16:0_16:0)	ND	4.70E+11	ND	ND	ND	ND	ND	ND	4.70E+11	ND	ND
TG(18:4_16:0_18:3)	ND	ND	ND	ND	1.93E+10	1.93E+10	3.08E+10	ND	1.93E+10	1.93E+10	3.08E+10
TG(18:4_16:1_18:4)	4.88E+11	ND	ND	ND	ND	ND	ND	4.88E+11	ND	ND	ND
TG(18:4_18:1_23:1)	ND	ND	ND	ND	ND	ND	4.77E+10	ND	ND	ND	4.77E+10
TG(18:4_18:3_18:3)	1.60E+12	ND	ND	ND	ND	ND	ND	1.60E+12	ND	ND	ND
TG(18:4_18:3_18:4)	7.06E+11	ND	ND	ND	ND	ND	ND	7.06E+11	ND	ND	ND
TG(18:4_18:3_22:6)	7.84E+09	ND	ND	ND	ND	ND	ND	7.84E+09	ND	ND	ND
TG(19:0_18:1_18:1)	ND	2.49E+12	ND	ND	3.82E+11	ND	ND	ND	2.87E+12	ND	ND
TG(19:1_18:1_18:1)	ND	ND	ND	ND	3.92E+11	ND	ND	ND	3.92E+11	ND	ND
TG(19:1_18:1_18:2)	ND	ND	ND	ND	2.85E+11	6.06E+11	4.63E+11	ND	2.85E+11	6.06E+11	4.63E+11
TG(19:1_18:1_20:4)	ND	ND	ND	ND	2.79E+10	4.96E+10	ND	ND	2.79E+10	4.96E+10	ND
TG(19:1_18:2_18:2)	ND	ND	ND	ND	ND	2.66E+11	1.95E+11	ND	ND	2.66E+11	1.95E+11
TG(20:0e_16:0_16:0)	ND	ND	ND	ND	ND	ND	3.29E+09	ND	ND	ND	3.29E+09
TG(20:1_18:1_18:1)	ND	2.01E+13	ND	ND	1.90E+12	2.59E+12	2.48E+12	ND	2.20E+13	2.59E+12	2.48E+12

TG(20:1_18:1_18:2)	ND	2.00E+13	ND	ND	2.48E+12	3.30E+12	3.52E+12	ND	2.25E+13	3.30E+12	3.52E+12
TG(20:1_18:1_20:1)	ND	ND	ND	ND	5.20E+11	ND	6.45E+11	ND	5.20E+11	ND	6.45E+11
TG(20:1_18:1_20:3)	ND	ND	ND	ND	ND	1.42E+11	1.08E+11	ND	ND	1.42E+11	1.08E+11
TG(20:1_18:1_20:4)	ND	ND	ND	ND	ND	1.37E+11	1.44E+11	ND	ND	1.37E+11	1.44E+11
TG(20:1_18:1_22:4)	ND	ND	ND	1.30E+12	1.63E+11	2.55E+11	2.47E+11	ND	1.63E+11	2.55E+11	1.55E+12
TG(20:1_18:1_22:5)	ND	ND	ND	ND	6.71E+10	2.69E+11	1.20E+11	ND	6.71E+10	2.69E+11	1.20E+11
TG(20:1_18:1_23:0)	ND	ND	ND	ND	9.26E+09	ND	ND	ND	9.26E+09	ND	ND
TG(20:1_18:2_22:5)	ND	ND	ND	ND	ND	1.83E+11	ND	ND	ND	1.83E+11	ND
TG(20:3_18:2_18:2)	ND	ND	ND	ND	6.57E+11	8.65E+11	1.08E+12	ND	6.57E+11	8.65E+11	1.08E+12
TG(20:3_18:2_20:5)	ND	ND	ND	ND	ND	5.54E+10	ND	ND	ND	5.54E+10	ND
TG(20:3_18:2_22:4)	ND	ND	ND	ND	ND	ND	5.66E+10	ND	ND	ND	5.66E+10
TG(20:3_18:2_22:6)	ND	ND	ND	ND	4.72E+10	7.97E+10	7.65E+10	ND	4.72E+10	7.97E+10	7.65E+10
TG(20:5_17:1_18:2)	ND	ND	ND	ND	ND	2.62E+10	ND	ND	ND	2.62E+10	ND
TG(20:5_18:2_22:4)	ND	ND	ND	ND	3.99E+10	6.05E+10	5.80E+10	ND	3.99E+10	6.05E+10	5.80E+10
TG(20:5_18:2_22:5)	ND	ND	7.66E+10	ND	ND	6.51E+10	7.04E+10	ND	ND	1.42E+11	7.04E+10
TG(20:5_18:2_22:6)	4.52E+10	ND	ND	ND	ND	ND	ND	4.52E+10	ND	ND	ND
TG(22:4_18:2_18:2)	ND	ND	ND	ND	3.65E+11	6.12E+11	6.25E+11	ND	3.65E+11	6.12E+11	6.25E+11
TG(22:5_18:2_20:4)	ND	6.72E+10	ND	ND	ND	ND	ND	ND	6.72E+10	ND	ND
TG(25:0_16:0_18:1)	ND	1.95E+11	ND	ND	3.36E+10	3.00E+10	4.15E+10	ND	2.29E+11	3.00E+10	4.15E+10
TG(26:0_18:1_18:2)	ND	ND	ND	ND	1.62E+10	ND	ND	ND	1.62E+10	ND	ND
TG(28:0_16:0_18:1)	ND	ND	ND	ND	4.55E+09	9.84E+09	ND	ND	4.55E+09	9.84E+09	ND
TG(4:0_14:0_14:0)	ND	ND	ND	ND	3.44E+09	ND	4.88E+09	ND	3.44E+09	ND	4.88E+09
TG(4:0_6:0_16:0)	ND	ND	ND	ND	ND	ND	1.04E+09	ND	ND	ND	1.04E+09
TG(4:0_6:0_18:0)	ND	ND	ND	ND	ND	ND	2.65E+08	ND	ND	ND	2.65E+08
TG(4:0_6:0_18:1)	ND	ND	ND	ND	ND	ND	4.57E+08	ND	ND	ND	4.57E+08
TG(6:0_12:0_18:1)	ND	ND	ND	ND	ND	1.29E+10	ND	ND	ND	1.29E+10	ND
TG(6:0_12:1_14:0)	ND	ND	ND	ND	1.97E+09	ND	2.57E+09	2.57E+09	1.97E+09	ND	2.57E+09
TG(6:0_12:1_18:1)	ND	ND	ND	ND	3.45E+10	1.98E+10	3.51E+10	3.51E+10	3.45E+10	1.98E+10	3.51E+10
TG(6:0_12:1_18:2)	ND	ND	ND	ND	1.51E+10	1.06E+10	2.71E+10	2.71E+10	1.51E+10	1.06E+10	2.71E+10
TG(6:0_12:2_18:2)	ND	ND	ND	ND	3.21E+09	ND	6.35E+09	6.35E+09	3.21E+09	ND	6.35E+09
TG(8:0_10:1_18:1)	ND	ND	ND	ND	ND	1.35E+10	3.10E+10	3.10E+10	ND	1.35E+10	3.10E+10
TG(8:0_8:0_10:0)	ND	ND	ND	ND	ND	5.73E+08	ND	ND	ND	5.73E+08	ND
WE(6:0_16:3)	3.48E+11	ND	5.77E+11	ND	ND	ND	ND	3.48E+11	ND	5.77E+11	ND

ND means not detected.

Supplementary Table 3. Typical MS2 data of some uncommon lipid ions in tilapia juice.

Steaming time point	LipidIon	ProductIon
10min	BisMePA(18:0_20:5)+NH4	C8H13(109.101532):MS2,C9H11(119.085723):MS2,C9H15(123.116634):MS2,C10H13(133.101354):MS2,C15H23(203.179232):MS2,[FA(18:0)-C3H4OH]+H(341.305022):MS2,[M+NH4]-NH3-HO-PO(OCH3)2-FA(20:5)ketene(341.305022):MS2
	BisMePA(34:1_16:0)+NH4	C8H11(107.08582):MS2,C8H13(109.101636):MS2,C9H11(119.085434):MS2,C9H15(123.117057):MS2,C10H13(133.101577):MS2,FA(16:0)-OH(239.236777):MS2,[FA(16:0)-C3H4OH]+H(313.274767):MS2,[M+NH4]-NH3-HO-PO(OCH3)2-FA(34:1)ketene(313.274767):MS2,[M+NH4]-NH3-FA(16:0)(671.534676):MS2
	CmE(2:0)+NH4	C8H11(107.085947):MS2,C8H13(109.101535):MS2,C9H13(121.101389):MS2,C9H15(123.116989):MS2,C10H15(135.116967):MS2,C10H17(137.132519):MS2,C11H15(147.11654):MS2,C11H17(149.132629):MS2,C12H17(161.132989):MS2,C13H19(175.14821):MS2
	TG(16:0_9:0_9:0)+NH4	C8H11(107.085836):MS2,C8H13(109.101563):MS2,C8H15(111.117279):MS2,C9H13(121.101272):MS2,C9H15(123.117093):MS2,C9H17(125.132522):MS2,C10H15(135.116956):MS2,C10H17(137.132631):MS2,FA(9:0)-OH(141.12779):MS2,C11H15(147.116744):MS2,C11H17(149.132843):MS2,C11H19(151.148533):MS2,C12H17(161.132908):MS2,C13H19(175.148542):MS2,FA(16:0)-OH(239.236595):MS2,MG(16:0)-OH(313.273717):MS2,NL[FA(16:0)-H+NH4](355.284168):MS2,M-OH(593.515982):MS2
	TG(4:0_14:0_14:0)+NH4	C8H13(109.101625):MS2,C9H13(121.100946):MS2,C9H15(123.117104):MS2,C10H17(137.132366):MS2,FA(14:0)-OH(211.206663):MS2,MG(14:0)-OH(285.242633):MS2,NL[FA(14:0)-H+NH4](355.286037):MS2
	TG(6:0_12:1_14:0)+NH4	FA(6:0)-OH(99.080858):MS2,C10H15(135.116555):MS2,MG(14:0)-OH(285.242138):MS2
30min	BisMePA(16:1e_16:0)+NH4	C8H13(109.101557):MS2,C9H15(123.11707):MS2,C10H13(133.100908):MS2,FA(16:0)-OH(239.23794):MS2,FA(16:0)+H(257.247601):MS2,[FA(16:0)-C3H4OH]+H(313.273438):MS2,[M+NH4]-NH3-HO-PO(OCH3)2-FA(16:1e)ketene(313.273438):MS2,[M+NH4]-NH3-FA(16:0)(405.276914):MS2
	BisMePA(16:1e_18:1)+NH4	C8H11(107.08585):MS2,C8H13(109.101524):MS2,C9H15(123.117086):MS2,C10H13(133.100994):MS2,FA(18:1)-OH(265.252049):MS2,[]FA(18:1)-C3H4O](337.273732):MS2,[M+NH4]-NH3-HO-PO(OCH3)2-FA(16:1e)ketene(339.289122):MS2,[FA(18:1)-C3H4OH]+H(339.289122):MS2,[M+NH4]-NH3-FA(18:1)(405.276433):MS2
	BisMePA(16:1e_18:2)+NH4	C8H11(107.08595):MS2,C8H13(109.101475):MS2,C9H11(119.085365):MS2,C9H15(123.117046):MS2,C10H13(133.101184):MS2,FA(18:2)-OH(263.237523):MS2,[FA(18:2)-C3H4OH]+H(337.273425):MS2,[M+NH4]-NH3-HO-PO(OCH3)2-FA(16:1e)ketene(337.273425):MS2,[M+NH4]-NH3-FA(18:2)(405.275645):MS2
	BisMePA(16:2e_18:2)+NH4	C8H9(105.070372):MS2,C8H11(107.086):MS2,C9H15(123.116797):MS2,C10H13(133.100634):MS2,[]FA(18:2)-C3H4O](335.256526):MS2,[FA(18:2)-C3H4OH]+H(337.273265):MS2,[M+NH4]-NH3-HO-PO(OCH3)2-FA(16:2e)ketene(337.273265):MS2,[M+NH4]-NH3-FA(18:2)(403.259622):MS2
	BisMePA(30:1_16:0)+NH4	C8H11(107.085915):MS2,C8H13(109.101496):MS2,C9H11(119.085993):MS2,C9H15(123.11715):MS2,C10H13(133.101534):MS2,FA(16:0)-OH(239.23679):MS2,[FA(16:0)-C3H4OH]+H(313.273565):MS2,[M+NH4]-NH3-HO-PO(OCH3)2-FA(30:1)ketene(313.273565):MS2,[M+NH4]-NH3-FA(16:0)(615.473425):MS2
	BisMePA(30:1_18:1)+NH4	C8H9(105.070348):MS2,C8H11(107.085974):MS2,C8H13(109.101623):MS2,C9H11(119.085537):MS2,C9H15(123.117392):MS2,C10H13(133.101225):MS2,FA(18:1)-OH(265.252512):MS2,[]FA(18:1)-C3H4O](337.274204):MS2,[M+NH4]-NH3-HO-PO(OCH3)2-FA(30:1)ketene(339.28846):MS2,[FA(18:1)-C3H4OH]+H(339.28846):MS2,[M+NH4]-NH3-FA(18:1)(615.473347):MS2
	BisMePA(30:1_18:2)+NH4	C8H9(105.070006):MS2,C8H13(109.101406):MS2,C9H11(119.085993):MS2,C9H15(123.117045):MS2,C10H11(131.08526):MS2,C10H13(133.101677):MS2,FA(18:2)-OH(263.236788):MS2,[FA(18:2)-C3H4OH]+H(337.272138):MS2,[M+NH4]-NH3-HO-PO(OCH3)2-FA(30:1)ketene(337.272138):MS2,[M+NH4]-NH3-FA(18:2)(615.473845):MS2
	BisMePA(32:1_16:0)+NH4	C8H9(105.070222):MS2,C8H13(109.101513):MS2,C9H11(119.085774):MS2,C9H15(123.117076):MS2,C10H13(133.101616):MS2,FA(16:0)-OH(239.237021):MS2,[FA(16:0)-C3H4OH]+H(313.274429):MS2,[M+NH4]-NH3-HO-PO(OCH3)2-FA(32:1)ketene(313.274429):MS2,[M+NH4]-NH3-FA(16:0)(643.504646):MS2,[M+NH4]-NH3-FA(16:0)[isotope](644.503491):MS2
	BisMePA(32:1_18:1)+NH4	C8H9(105.070266):MS2,C8H11(107.08589):MS2,C8H13(109.101527):MS2,C9H11(119.085897):MS2,C9H15(123.117009):MS2,C10H13(133.10087):MS2,FA(18:1)-OH(265.252589):MS2,[]FA(18:1)-C3H4O](337.27213):MS2,[M+NH4]-NH3-HO-PO(OCH3)2-FA(32:1)ketene(339.288623):MS2,[FA(18:1)-C3H4OH]+H(339.288623):MS2,[M+NH4]-NH3-FA(18:1)(643.505037):MS2
	BisMePA(32:1_18:2)+NH4	C8H9(105.070302):MS2,C8H11(107.085973):MS2,C8H13(109.101562):MS2,C9H11(119.085979):MS2,C9H15(123.117086):MS2,C10H11(131.085525):MS2,C10H13(133.101264):MS2,FA(18:2)-OH(263.237173):MS2,[]FA(18:2)-C3H4O](335.2592):MS2,[FA(18:2)-C3H4OH]+H(337.272092):MS2,[M+NH4]-NH3-HO-PO(OCH3)2-FA(32:1)ketene(337.272092):MS2,[M+NH4]-NH3-FA(18:2)(643.504515):MS2
	BisMePA(32:1_18:3)+NH4	C8H9(105.07026):MS2,C8H11(107.086057):MS2,C8H13(109.101565):MS2,C9H11(119.085823):MS2,C9H15(123.117057):MS2,C10H11(131.08543):MS2,C10H13(133.101411):MS2,FA(18:3)-OH(261.221215):MS2,FA(18:3)+H(279.232996):MS2,[M+NH4]-NH3-HO-PO(OCH3)2-FA(32:1)ketene(335.257789):MS2,[FA(18:3)-C3H4OH]+H(335.257789):MS2,[M+NH4]-NH3-FA(18:3)(643.503445):MS2
	BisMePA(34:1_18:1)+NH4	C8H9(105.070205):MS2,C8H11(107.085979):MS2,C8H13(109.101578):MS2,C9H11(119.085651):MS2,C9H15(123.11704):MS2,C10H11(131.085642):MS2,C10H13(133.101398):MS2,C15H23(203.179536):MS2,FA(18:1)-OH(265.252488):MS2,[M+NH4]-NH3-HO-PO(OCH3)2-FA(34:1)ketene(339.288539):MS2,[FA(18:1)-C3H4OH]+H(339.288539):MS2,[M+NH4]-NH3-FA(18:1)(671.535822):MS2
	BisMePA(34:1_18:2)+NH4	C8H11(107.085818):MS2,C8H13(109.101591):MS2,C9H11(119.085789):MS2,C9H15(123.117091):MS2,C10H11(131.085938):MS2,C10H13(133.101318):MS2,FA(18:2)-OH(263.236978):MS2,[FA(18:2)-C3H4OH]+H(337.274496):MS2,[M+NH4]-NH3-HO-PO(OCH3)2-FA(34:1)ketene(337.274496):MS2,[M+NH4]-NH3-FA(18:2)(671.535232):MS2

Cer(d16:2_21:1)+CH3COO	FAmide(21:1)-CH2-H(308.296179):MS2,FAmide(21:1)-H(338.307623):MS2,M-H-H2-H2CO(542.493236):MS2,M-H-H2CO(544.513068):MS2,M-H(574.521744):MS2
TG(14:1e_10:1_10:1)+H	C7H11(95.08586):MS2,C8H11(107.085933):MS2,C8H13(109.101546):MS2,C9H13(121.101302):MS2,C9H15(123.116805):MS2,C10H15(135.116943):MS2,C10H17(137.132543):MS2,C11H15(147.116795):MS2, C11H17(149.132631):MS2,C11H19(151.14827):MS2,FA(10:1)-OH(153.127591):MS2,C18H27(243.210808):MS2,NL[FA(10:1)-FA(10:1)](251.236839):MS2,M+H(591.496823):MS2
WE(6:0_16:3)+H	prod(57.0705(57.0707):MS2,C5H7(67.054877):MS2,C5H9(69.070561):MS2,C5H11(71.086191):MS2,C6H9(81.070504):MS2,C6H11(83.086054):MS2,C6H13(85.101911):MS2,C7H11(95.085955):MS2,C7H13(97.101712):MS2,C8H13(109.101529):MS2,C8H15(111.116755):MS2,C9H15(123.116995):MS2,C10H17(137.132372):MS2,C11H15(147.116861):MS2,C11H19(151.14777):MS2,C12H17(161.132042):MS2,C13H19(175.147123):MS2,FA(16:3)+H(251.200699):MS2,M+H(335.29311):MS2

Supplementary Table 4. Lipid species in muscles and juices during the tilapia muscle steaming processes

	0 min	10 min	30 min	60 min
Muscle	AcCa, Cer, CL, DG, LPC, LPI, LPS, PC, PE, PG, PI, TG, WE	Cer, CL, FA, LPC, LPG, LPI, LPS, PC, PE, PI, SM, TG	Cer, dMePE, FA, LPC, LPE, LPG, LPI, MG, PC, PE, PI, PS, TG, WE	Cer, CL, FA, LPC, LPE, LPG, LPI, LPS, PC, PE, TG
Juice	NA	BisMePA, Cer, CmE, DG, FA, LPC, PC, PE, PEt, PG, PI, PS, SM, SPH, TG	BisMePA, Cer, DG, LPC, PC, PE, PEt, PG, PI, PS, SPH, TG	BisMePA, Cer, DG, FA, LPC, PC, PE, PEt, PG, PI, PS, SPH, TG
Presence in both muscle and juice at the same time points	NA	Cer, FA, LPC, PC, PE, PI, SM, TG	Cer, LPC, PC, PE, PI, PS, TG	Cer, FA, LPC, PC, PE, TG
Presence in both muscles and juice at all the time points	Cer, LPC, PC, PE, TG			

NA means not applicable.

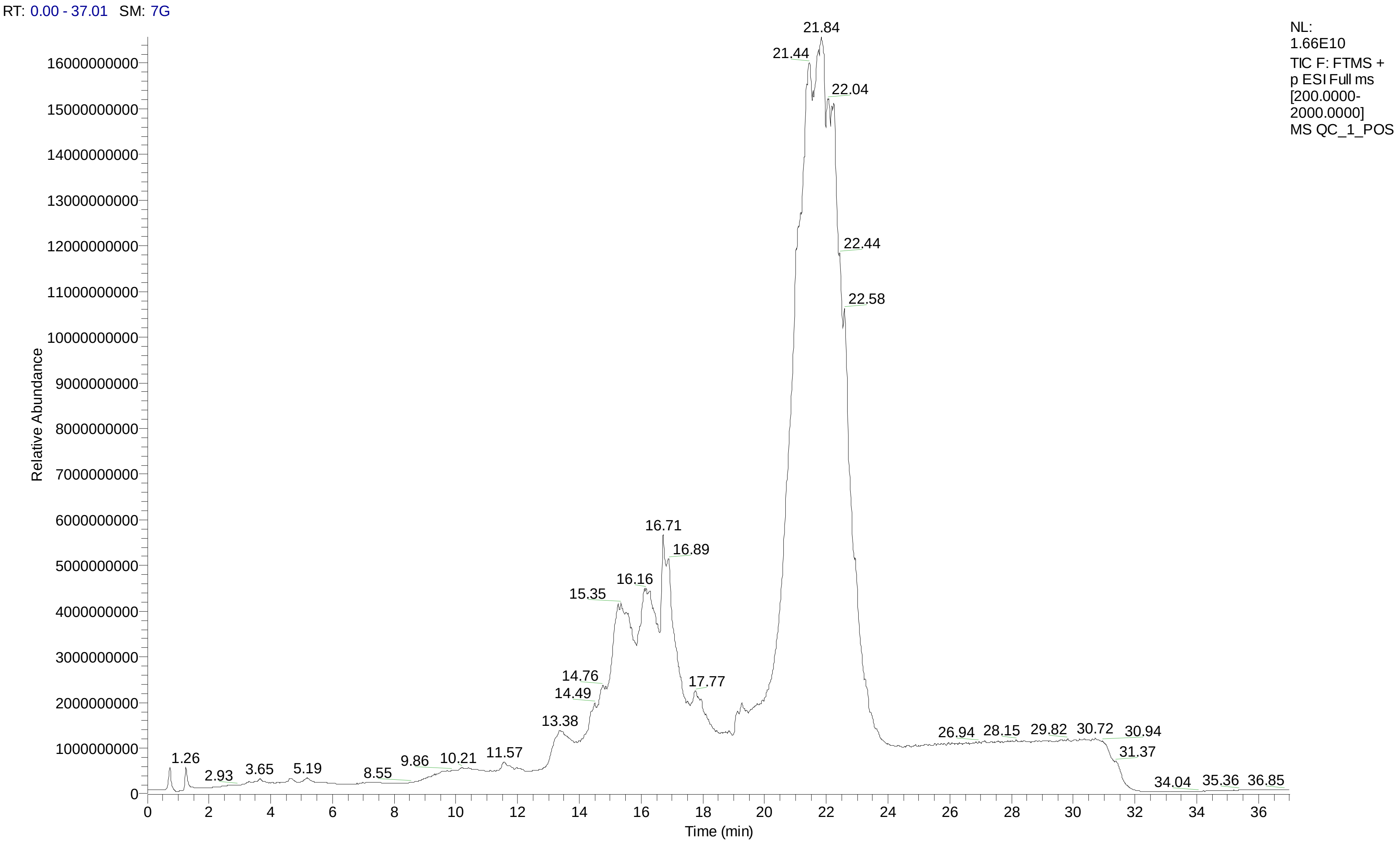

Supplementary Figure 1. UHPLC-Extractive Orbitrap MS base peak intensity chromatograms acquired in positive ionization mode of quality control sample.

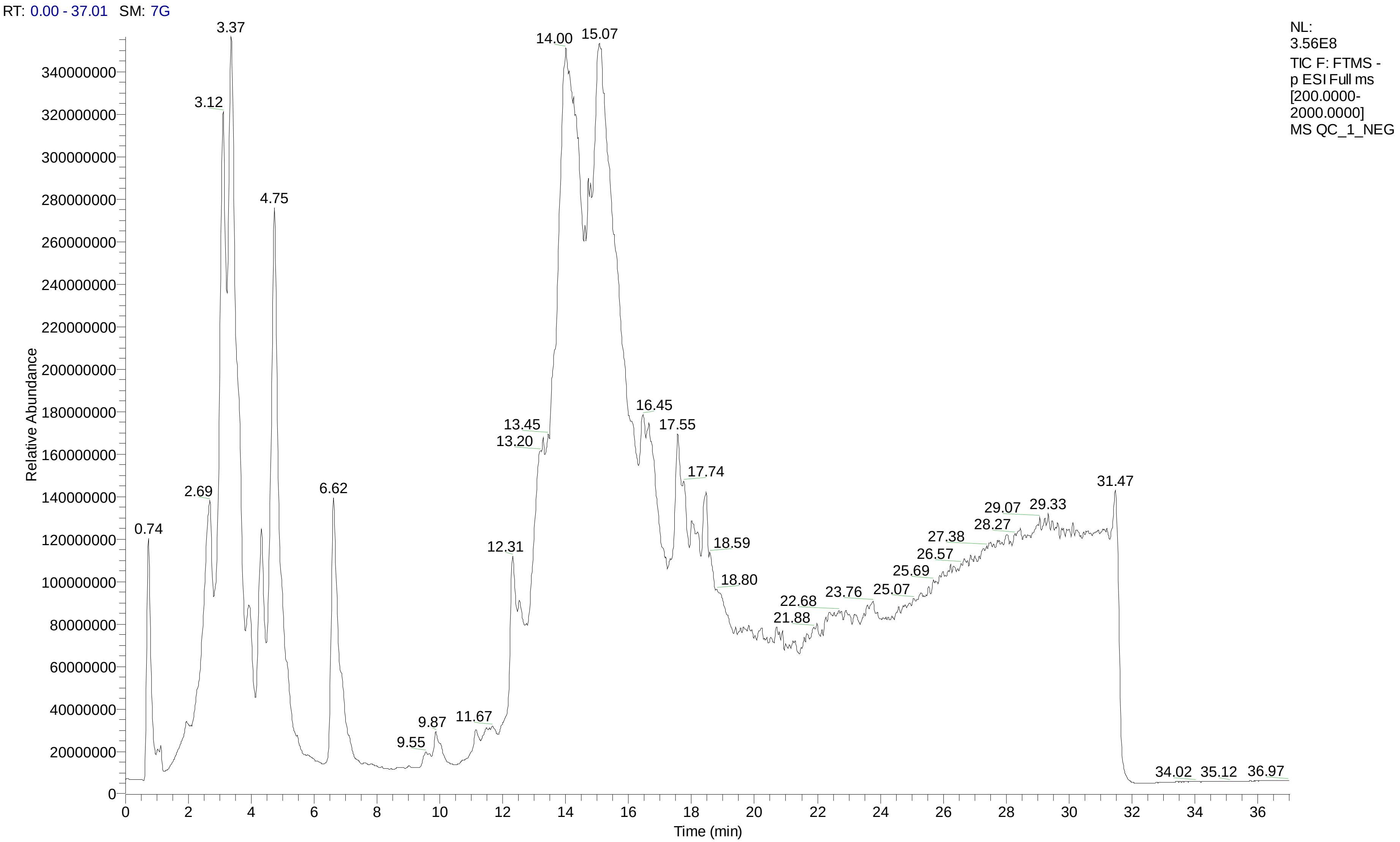

Supplementary Figure 2. UHPLC-Extractive Orbitrap MS base peak intensity chromatograms acquired in negative ionization mode of quality control sample.

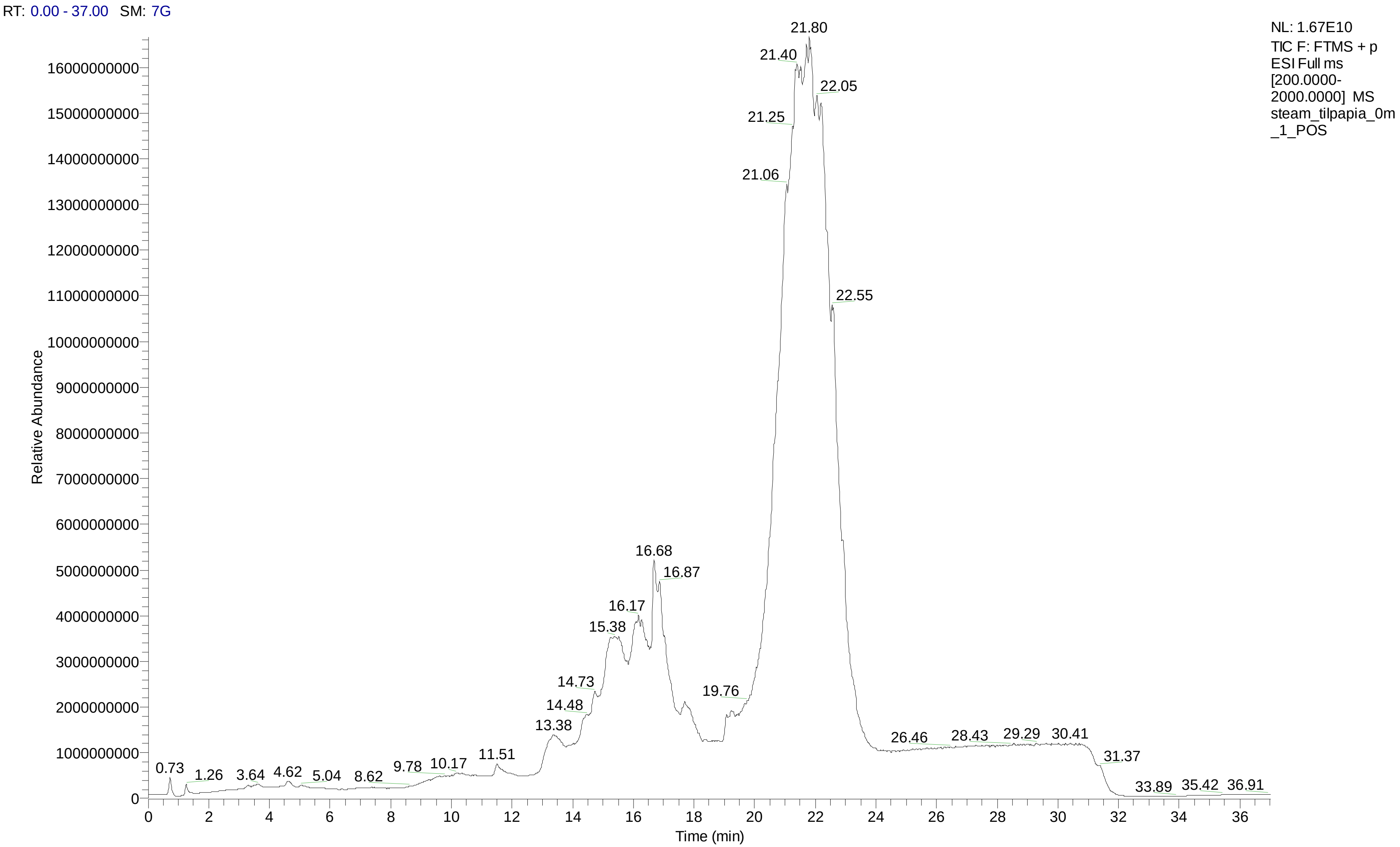

Supplementary Figure 3. UHPLC-Extractive Orbitrap MS base peak intensity chromatograms acquired in positive ionization mode of tilapia muscle at 0 min.

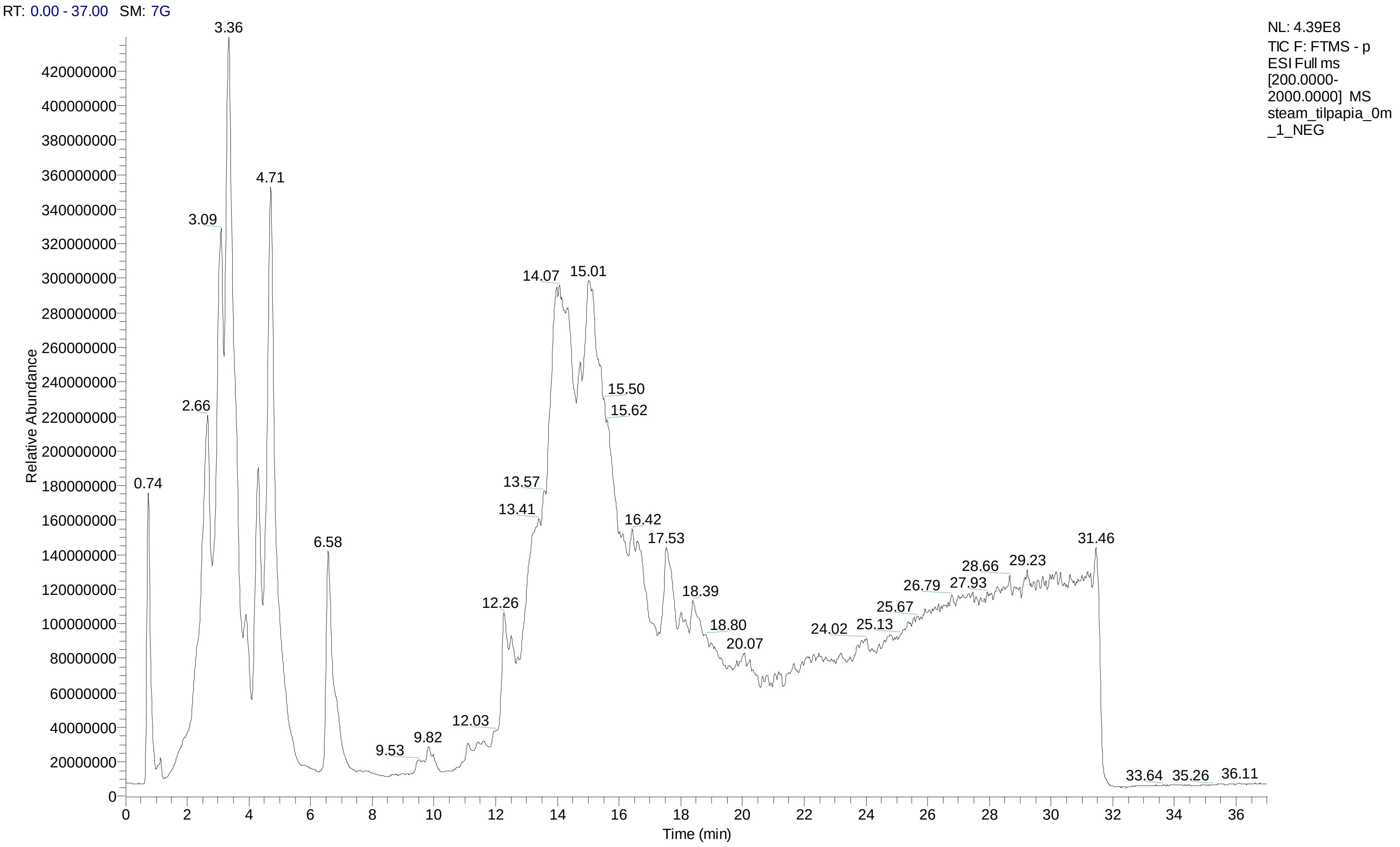

Supplementary Figure 4. UHPLC-Extractive Orbitrap MS base peak intensity chromatograms acquired in negative ionization mode of tilapia muscle at 0 min.
